# Multi-epigenome-wide analyses and meta-analysis of child maltreatment in judicial autopsies and intervened children and adolescents

**DOI:** 10.1038/s41380-025-03236-1

**Published:** 2025-09-16

**Authors:** Shota Nishitani, Takashi X. Fujisawa, Shinichiro Takiguchi, Akiko Yao, Kazuhiro Murata, Daiki Hiraoka, Yoshifumi Mizuno, Keiko Ochiai, Natasha Y. S. Kawata, Kai Makita, Daisuke N. Saito, Sakae Mizushima, Shizuka Suzuki, Sawa Kurata, Naoki Ishiuchi, Daiki Taniyama, Naoki Nakao, Akira Namera, Hidehiko Okazawa, Masataka Nagao, Akemi Tomoda

**Affiliations:** 1 Research Center for Child Mental Development, University of Fukui, Fukui, Japan; 2 Division of Developmental Higher Brain Functions, United Graduate School of Child Development, Osaka University, Kanazawa University, Hamamatsu University School of Medicine, Chiba University, and University of Fukui, Osaka, Japan; 3 Life Science Innovation Center, School of Medical Sciences, University of Fukui, Fukui, Japan; 4 Department of Child and Adolescent Psychological Medicine, University of Fukui Hospital, Fukui, Japan; 5 Department of Forensic Medicine, Graduate School of Biomedical and Health Sciences, Hiroshima University, Hiroshima, Japan; 6 Center for Cause of Death Investigation Research Graduate School of Biomedical and Health Sciences, Hiroshima University, Hiroshima, Japan; 7 Department of Science of Human Development, Faculty of Education, Humanities and Social Sciences, University of Fukui, Fukui, Japan; 8 Department of Molecular Pathology, Graduate School of Biomedical and Health Sciences, Hiroshima University, Hiroshima, Japan; 9 Biomedical Imaging Research Center, University of Fukui, Fukui, Japan

**Keywords:** Predictive markers, Neuroscience

## Abstract

Child maltreatment (CM) is associated with adverse physical, psychological, and neurodevelopmental outcomes later in life. Epigenetic modifications, particularly DNA methylation, have been proposed as potential mechanisms underlying these long-term effects. To identify robust CM-associated methylation signatures, we conducted epigenome-wide analyses across three independent cohorts: judicial autopsy cases (CM:11, Controls:7), toddlers shortly after social intervention (CM:36, Controls:49), and adolescents who underwent brain MRI (CM:61, Controls:62). Each cohort was analyzed separately, followed by a meta-analysis to identify common methylation sites associated with CM exposure. The meta-analysis identified four significant CpG sites located within the *ATE1*, *SERPINB9P1*, *CHST11*, and *FOXP1* genes. Among these, methylation of *FOXP1* was consistently associated with structural brain alterations, including increased gray matter volume (GMV) in the orbitofrontal cortex (OFrC) and middle/posterior cingulate gyrus (MPCG), and decreased GMV in the occipital fusiform gyrus (OFuG). These brain regions are implicated in emotional regulation, memory retrieval, and social cognition, suggesting a potential neurobiological mechanism linking CM to later psychopathology. Furthermore, methylation risk scores (MRS) derived from these four CpGs successfully discriminated individuals who experienced early-life adversity in an independent validation dataset, achieving an area under the receiver operating characteristic curve (AUC) of 0.672, highlighting their potential utility as biomarkers. Gene ontology and pathway analyses revealed enrichment of cholinergic and glutamatergic synaptic transmission pathways, supporting their involvement in traumatic memory formation. Our findings provide novel insights into the epigenetic mechanisms underlying CM and identify potential biomarkers for early detection, prevention, and therapeutic intervention, ultimately contributing to breaking the intergenerational cycle of maltreatment.

## Introduction

Child maltreatment (CM), including abuse and neglect, profoundly impacts children’s physical and mental health, increasing risks for psychiatric disorders, cardiovascular diseases, and suicide [1]. Epigenetic mechanisms, particularly DNA methylation, have been proposed as mediators linking early-life adversity to long-term health outcomes. Such epigenetic alterations may leave specific molecular signatures in children exposed to CM [2]. Consequently, numerous epigenetic studies have aimed not only to elucidate underlying biological mechanisms but also to identify biomarkers for early detection and prevention of CM.

However, most previous epigenetic studies have employed candidate-gene approaches based on specific hypotheses, such as stress-related [3] or sociability-related genes [4, 5]. These studies were not designed to discover novel or unexpected epigenetic markers beyond existing knowledge. Therefore, epigenome-wide association studies (EWAS) are essential for comprehensively identifying DNA methylation alterations associated with CM. Although promising, many prior EWAS have been conducted in adults retrospectively reporting childhood maltreatment, rather than in children themselves [6]. Such retrospective studies have limited utility for developing preventive biomarkers due to the substantial time gap between maltreatment exposure and methylation assessment. Additionally, methylation changes identified in adults may not reflect dynamic responses directly attributable to CM, as intervening environmental factors could confound results.

Conducting EWAS in children presents unique ethical and practical challenges. Obtaining informed consent from minors is complicated, particularly given sensitive family backgrounds and legal considerations. Consequently, only a limited number of epigenetic studies have directly investigated children exposed to maltreatment. To date, fewer than ten EWAS have examined DNA methylation in children close to the time of maltreatment exposure [7–13]. However, these studies have limitations, such as lack of functional interpretation [13], reliance on candidate genes [7], qualitative analyses [11], or small sample sizes [8]. A recent prospective study identified methylation sites dynamically altered by childhood adversity and highlighted sensitive developmental periods [9]. However, reliance on maternal self-reports may underestimate CM prevalence. Thus, further studies using cohorts with objectively verified CM exposure are necessary to confirm and extend these findings.

To address these gaps, we conducted EWAS in three independent cohorts comprising judicially or socially certified CM cases and matched controls. Our primary aim was to identify robust epigenetic signatures associated with CM. We conducted EWAS separately in each cohort, followed by meta-analysis to identify common methylation markers. We further examined associations between these markers and CM-related biological indicators, and evaluated their utility in classifying CM exposure. To our knowledge, this is the first EWAS of CM employing multiple independent cohorts analyzed in parallel using a unified analytical pipeline, enabling subsequent meta-analysis and synthesis of findings. Our study thus provides a critical step toward identifying reliable epigenetic biomarkers for early detection and prevention of CM, overcoming limitations of previous research.

## Methods

### Participants and cohorts

This study included three Japanese cohorts comprising a total of 226 children for genome-wide DNA methylation analyses (Table 1). The cohorts consisted of judicial autopsy cases, children sheltered in residential childcare facilities, and typically developing (TD) children raised by biological families recruited from the local community as controls. Children in residential childcare facilities had been legally removed from their biological parents by Child Protection Services or equivalent authorities, and most had documented histories of physical, emotional, or sexual abuse, or neglect prior to placement. Participants with documented maltreatment histories were classified as the CM group (ICD-10-CM Code T74). Psychosocial difficulties and depressive symptoms were assessed using the Strengths and Difficulties Questionnaire (SDQ) [14] and the Depression Self-Rating Scale for Children (DSRS-C) [15], respectively.

**Table 1:** Demographic characteristics of the three cohorts.

	CM	TD	*P-*value	Total
Number				
Judicial	11	7	–	18
Toddler	36	49	–	85
Adolescent	61	62	–	123
Age, mean (SD)				
Judicial	3.1 (3.4)	4.7 (7.4)	0.53	3.7 (5.2)
Toddler	5.8 (2.6)	4.4 (1.9)	0.009	5.0 (2.3)
Adolescent	13.5 (2.7)	14.1 (2.9)	0.19	13.8 (2.8)
Sex (Male/Female)				
Judicial	4 / 7	2 / 5	0.73	6 / 12
Toddler	21 / 15	26 / 23	0.63	47 / 38
Adolescent	38 / 23	38 / 24	0.91	76 / 47
IQ / DQ, mean (SD)				
Toddler	88.3 (12.5)	102.8 (9.6)	4.0E-08	96.7 (13.1)
Adolescent	91.5 (11.7)	108.1 (10.6)	1.7E-13	99.9 (13.9)
SDQ, mean (SD)				
Toddler^a^	11.8 (6.5)	7.5 (4.7)	0.0015	9.4 (5.9)
Adolescent^b^	11.7 (5.7)	5.9 (3.9)	2.3E-09	8.6 (5.7)
DSRS-C, mean (SD)				
Adolescent^c^	11.9 (7.2)	7.3 (4.8)	6.9E-05	9.5 (6.5)
Maltreatment history				
Toddler				
PA (%)	6 (16.7)	–	–	–
EA (%)	17 (47.2)	–	–	–
SA (%)	0 (0)	–	–	–
NG (%)	30 (83.3)	–	–	–
#Types, mean (SD)	1.5 (0.8)	–	–	–
Duration (y), mean (SD)	2.2 (2.0)	–	–	–
Adolescent				
PA (%)	36 (59.0)	–	–	–
EA (%)	37 (60.7)	–	–	–
SA (%)	5 (8.2)	–	–	–
NG (%)	49 (80.3)	–	–	–
#Types, mean (SD)	2.1 (0.9)	–	–	–
Duration (y), mean (SD)	7.1 (3.7)	–	–	–

The *P*-values were computed using *t*-test (for continuous variables) and *χ2* test (for categorical variables) for comparison of CM and TD groups.

*PA* physical abuse, *EA* emotional abuse, *SA* sexual abuse, *NG* neglect, *SDQ* Strength and Difficulties Questionnaire, *DSRS-C* depression self-rating scale for children,

^a^Missing data for CM (3), and TD (7),

^b^Missing data for CM (5),

^c^Missing data for CM (5).

### Judicial autopsy cases

Twenty-six children whose deaths were judicially authenticated by a forensic pathologist (M.N.) between 2000 and 2021 were included. Of these, 15 cases (CM) had causes of death attributed to child abuse or neglect, and the remaining 11 cases (TD) were due to fatal accidents or illnesses (Supplementary Table S1). Thymus weight records were available for 24 cases (CM:15, TD:9). Whole blood samples from 18 cases (CM:11, TD:7) had been stored at −20 °C. Formalin-fixed paraffin-embedded (FFPE) brain tissues or formalin-immersed brain blocks were preserved for 24 cases (CM:13, TD:11). Prefrontal cortex tissues were selected for methylation analysis, as this region was consistently preserved across all cases. The study protocol was approved by the Ethics Committee of the University of Fukui (approval no. 20200030) and the Research Ethics Review Board of Hiroshima University (approval no. E–2032), and was conducted in accordance with the Declaration of Helsinki.

### Toddler social cognition cohort

One hundred twenty-two children aged 0–9 years participated in this cohort between 2017 and 2021. Participants underwent assessments of social cognitive function using gaze pattern analysis and provided buccal mucosa samples [16–18]. Genome-wide methylation analysis was conducted on 85 participants (CM:36, TD:49) who passed the quality control (QC) procedures described below, had no repeated measurements, and completed cognitive assessments using either the Wechsler Intelligence Scale for Children-Fourth Edition (WISC-IV), Kyoto Scale of Psychological Development (KSPD), or equivalent developmental scales (Table 1). The study protocol was approved by the Ethics Committee of the University of Fukui (approval nos. 20140142, 20150068, and 20190107) and conducted in accordance with the Declaration of Helsinki. Written informed consent was obtained from all parents or childcare facility directors.

### Adolescent brain imaging cohort

Two hundred thirty-seven children and adolescents aged 6–18 years (CM:83, TD:154) participated in this cohort between 2013 and 2022, undergoing brain MRI scans [4, 5, 19–22] (Supplementary Table S2). Group comparisons of brain gray matter (GM) structures were conducted using the full dataset. Saliva samples were collected from 141 participants. Genome-wide methylation analysis was performed on a subset of 123 participants (CM:61, TD:62) who passed the QC procedures described below, had no repeated measurements, and completed full-scale IQ (FSIQ) assessments (Table 1). The study protocol was approved by the Ethics Committee of the University of Fukui (approval nos. 20110104, 20130157, 20138031, 20150068, 20190107, 20210004, 20220034, and 20220039) and conducted in accordance with the Declaration of Helsinki. Written informed consent was obtained from all parents or childcare facility directors.

### Sample collection and DNA extraction

For the Judicial Autopsy Cases, DNA was extracted from whole blood samples using the AllPrep DNA/RNA/miRNA Universal Kit (QIAGEN, Venlo, Netherlands). Brain DNA was extracted from FFPE tissues using the High Pure FFPET DNA Isolation Kit (Roche, Basel, Switzerland), starting from approximately 10 mg tissue blocks or six slices (12 µm thickness, 3 cm²) until a total yield of 250 ng DNA was obtained. A modified pre-processing protocol was employed to improve extraction efficiency, including two five-minute ethanol washes, an overnight PBS wash (50 °C, 600 rpm), and overnight lysis (56 °C, 600 rpm) [23, 24].

In the Toddler Social Cognition cohort, buccal swab samples were collected using commercially available cotton swabs, with either one swab (CM:13, TD:16) or four swabs (CM:23, TD:33) per individual [16, 18]. DNA was extracted using the QIAamp DNA Mini Kit (QIAGEN, Venlo, Netherlands). The first 16 TD swabs had unintentionally been stored at room temperature for an average of 461 ± 25 (SD) days before DNA extraction; thus, storage duration was included as a covariate in subsequent analyses. In the Adolescent Brain Imaging cohort, saliva samples were collected using the Oragene Discover OGR-500 kit (DNA Genotek Inc., Ottawa, Canada), and DNA was extracted using the prepIT®·L2P reagent (DNA Genotek) [4, 5, 25]. A total of 119 individuals (CM:58, TD:61) underwent both brain MRI and saliva collection and were available for imaging epigenetics analysis. Within this group, 72 (CM:38, TD:34) saliva samples were collected on the day of brain imaging or within several days; however, the remaining 47 (CM:20, TD:27) samples showed discrepancies in the dates of collection. DNA concentration was quantified using the Qubit dsDNA HS Assay Kit (Thermo Fisher Scientific Inc., Pittsburgh, PA, USA).

### Quality control (QC) procedures for DNA methylation

Genome-wide DNA methylation was assessed using the Infinium HumanMethylationEPIC BeadChip Kit (Illumina). DNA samples (500 ng for peripheral and 250 ng for brain tissues) were bisulfite-converted using the EZ DNA Methylation™ Kit (Zymo Research). Standardized QC procedures for peripheral tissues (blood, buccal mucosa, and saliva) were conducted separately for each cohort, including rigorous probe and sample filtering, data normalization, and correction for batch effects (see Supplementary Methods). For brain samples, customized QC criteria were applied to accommodate variations in sample quality (see Supplementary Methods).

### Autopsied thymus weight ratio calculation

Thymus weight ratio, reflecting the severity and duration of child abuse or neglect [26], was calculated relative to age-specific normal ranges established by the Medico-Legal Society of Japan [27] (see Supplementary Methods).

### Assessment of social cognitive function by gaze patterns for toddler social cognition cohort

Social cognitive function was assessed using gaze fixation time on the eye region measured by the eye-tracking system (Gazefinder®), as previously described [17, 18, 28] (see Supplementary Methods).

### Brain voxel-based morphometry (VBM)

Structural MRI data were acquired from 237 participants using 3-Tesla scanners, and images were preprocessed using SPM12. Regional differences in gray matter volume (GMV) between groups were analyzed using VBM, adjusting for age, FSIQ, scanner type, and total GMV (see Supplementary Methods).

### Statistical analysis for genome-wide methylation profiling

Two sets of analyses—differentially methylated probe (DMP) analysis and Gene-based Association test for Multiple Traits (GAMuT)—were performed for each cohort to identify methylation and gene associated with CM.

For the DMP analysis, we applied a multiple linear regression model using the *limma* package [29], with DNA methylation as the dependent variable and group (CM vs. TD) as the independent variable, adjusting for relevant covariates. Specifically, covariates included age, sex, days after death, and estimated cell-type proportions (CD8T, CD4T, NK, B, and monocytes) for the Judicial Autopsy Cases; age, sex, IQ or DQ, days until DNA extraction, and buccal cell proportions for the Toddler Social Cognition cohort; and age, sex, FSIQ, and buccal cell proportions for the Adolescent Brain Imaging cohort. Genome-wide analyses often exhibit inflation (reflected by an increased λ), typically due to residual confounding factors, leading to inflated type I error rates and false-positive findings. To correct for this inflation, we applied the *bacon* method [30] to the DMP analysis results. A Storey’s *q*-value < 0.05 was considered statistically significant. For the top 20 CpGs identified in these DMP analyses, we conducted association analyses with the endophenotypes and clinical measures described above for each cohort.

The GAMuT is a recently developed genome-wide analytical method designed to examine epigenetic associations across multiple phenotype domains simultaneously [31]. In this study, we conducted gene-based analyses to estimate epigenetic associations across maltreatment-type domains. Each CpG site was assigned to its closest gene using *hiAnnotator* [32] and Ensembl gene predictions, following the approach described by Hüls et al. [31]. All CpG sites within 20 kb of the nearest gene were included. In addition to the group variable, each maltreatment domain (physical abuse [PA], emotional abuse [EA], sexual abuse [SA], and neglect [NG]) was tested individually or aggregated into a composite maltreatment measure. Covariates included in the GAMuT model were identical to those used in the DMP analyses. To control for multiple testing, we adopted a significance threshold of *P* < 5.0E-05, consistent with the suggestive threshold used by Hüls et al. [31]. Genes exceeding this threshold in any group, composite measure, or individual maltreatment domain were identified. As a secondary analysis, we performed DMP analyses for each probe within these significant genes, testing associations with group and individual maltreatment domains. Probes with *P* < 0.001 in at least one domain were considered significant, following previously established criteria [31]. GAMuT analyses were not conducted for the Judicial Autopsy Cases, as multiple-trait maltreatment information was unavailable.

For brain methylation data, multiple regression analyses were performed individually for each probe meeting the customized QC criteria, using a model similar to that described above for blood methylation, except without adjustment for cell-type proportions.

### Meta-analysis

A meta-analysis was conducted on 816,366 CpGs common to all three cohorts using the weighted sum of *z*-scores method [33]. Multiple testing correction was performed using Storey’s *q*-value. To assess potential heterogeneity across cohorts, we calculated heterogeneity metrics (Cochran’s *Q*-statistic and *I²*) for significant CpG sites identified in the meta-analysis. For the CpGs identified as significant in the meta-analysis, we conducted association analyses with maltreatment history (described in detail below), as well as with the endophenotypes and clinical measures described above for each cohort.

### Enrichment analysis

To obtain a comprehensive overview of biological functions enriched among the top-ranked CpGs identified in the meta-analysis, Gene Ontology (GO) and Kyoto Encyclopedia of Genes and Genomes (KEGG) pathway enrichment analyses were conducted on the top 1500 CpG sites. GO enrichment results were visualized using a GO semantic similarity matrix [34], clustering significantly enriched GO terms (*P* < 0.05). KEGG pathways with *P* < 0.05 were considered significant.

### Evaluation of candidate CpGs from previously published studies

Candidate CpGs previously reported in five independent EWAS examining associations with CM [8, 10–13] were evaluated within the analytical framework of the meta-analysis.

### Maltreatment history

Because maltreatment history variables (age at exposure, type, and duration of maltreatment) inherently presume the presence of CM, analyses examining their associations were restricted to the CM-associated CpGs identified in our meta-analysis. To evaluate sensitive developmental periods and the relative importance of maltreatment types, we conducted random forest regression analyses as previously described [4] (see Supplementary Methods).

### Validation analysis

Validation analyses were conducted using the publicly available dataset comprises blood DNA methylation data (EPIC array) from institutionalized and family-raised children (GSE118940) [10]. Logistic regression and methylation risk scores (MRS) [35] were used to evaluate the predictive utility of the identified CpGs (see Supplementary Methods).

### Brain-peripheral DNA methylation correlations

To assess correlations between peripheral tissue methylation and brain methylation, the DMPs identified in each cohort and the meta-analysis were cross-referenced with the AMAZE-CpG database [36], which we previously developed for Japanese and Asian populations. This database provides Spearman’s correlation coefficients (*rho*) and associated *P*-values calculated from paired samples of live brain and peripheral tissues (blood, buccal, and saliva) obtained from 19 Japanese individuals.

## Results

### Demographic characteristics of participants

Demographic data for all participants are summarized in Table 1. Age differed significantly between CM and TD groups only in the Toddler Social Cognition cohort. Sex distribution did not differ significantly between groups in any cohort. Age and sex were included as covariates in all genome-wide analyses, and IQ/DQ was additionally included as a covariate in the Toddler Social Cognition and Adolescent Brain Imaging cohorts (see Supplementary Results). The CM group exhibited significantly higher SDQ and DSRS-C compared to the TD group.

### Judicial autopsy cases (CM:N = 11, TD:N = 7)

**Fig. 1:**
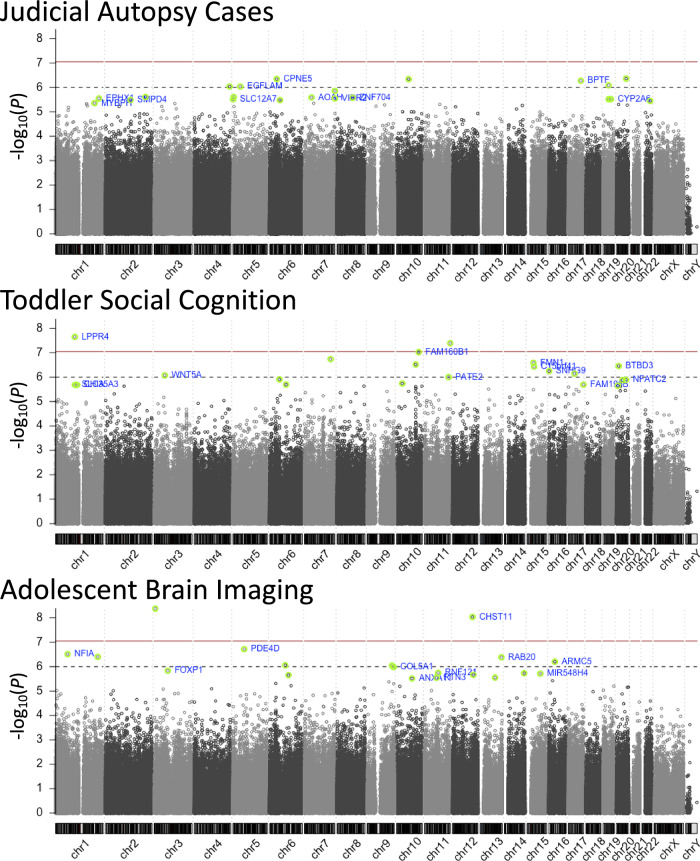
Manhattan plots for Judicial Autopsy Cases, Toddler Social Cognition, and Adolescent Brain Imaging cohorts. The horizontal solid line (dark red) indicates *P* = 9.0E-08, and the dashed line (gray) indicates *P* = 1.0E-06. The top 20 probes are highlighted in light green and labeled with their corresponding gene names. For details on the *bacon* adjustment and evaluation results regarding the overall quality of the genome-wide analysis (including Q-Q plots and genomic inflation factors), see Supplementary fig. S2.

**Fig. 2:**
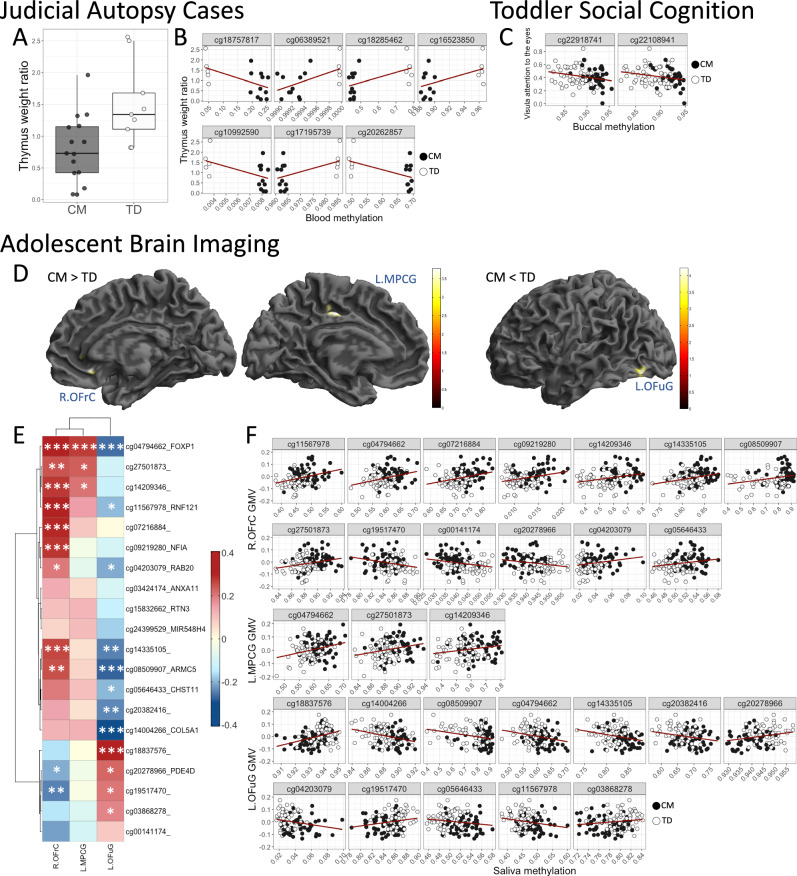
Results of analyzing differences in endophenotypes across each cohort, together with the significant associations between the top 20 DMPs identified in each EWAS and each endophenotype. **Judicial Autopsy Cases:** (**A**) Group comparison of thymus weight ratio (*t* = −2.9, *P* = 0.008). Error bars represent SEM. **B** Correlation plots for the seven DMPs that showed significant associations with thymus weight ratio. See Supplementary Table S3 for detailed statistical analyses. **Toddler Social Cognition:** (**C**) Correlation plots for the two DMPs significantly associated with visual attention to the eyes. See Supplementary Table S5 for full statistical details. **Adolescent Brain Imaging:** (**D**) Whole-brain GMV comparison using Voxel-Based Morphometry (VBM). The three identified brain regions are R.OFrC (Right Orbitofrontal Cortex), L.MPCG (Left Medial/Posterior Cingulate Gyrus), and L.OFuG (Left Occipital Fusiform Gyrus). Color bars indicate *t*-statistics. **E** Heatmap showing correlations between the top 20 DMPs and the GMVs of these three regions. Asterisks denote significance levels (*P < 0.05, **P < 0.01, ***P < 0.005). **F** Correlation plots for the DMPs significantly associated with each regional GMV: 13 (R.OFrC), 3 (L.MPCG), and 12 (L.OFuG). See Supplementary Table S8 for detailed statistical analyses.

No significant DMPs were identified after correcting for inflation (Fig. 1, Supplementary fig. S2A, and Supplementary Table S3). The CM group exhibited a significantly lower thymus weight ratio compared to the TD group (*t* = −2.9, *df* = 22, *P* < 0.01) (Fig. 2A). Among the top 20 DMPs, seven CpGs showed significant correlations with thymus weight ratio (Fig. 2B), and two CpGs (cg22646780 and cg16523850) also showed significant group differences in brain methylation data (see Supplementary Results and Supplementary Table S3). The full brain methylation results are presented in Supplementary Table S4.

### Toddler social cognition cohort (CM:N = 36, TD:N = 49)

Nine significant DMPs were identified after correcting for inflation (Fig. 1, Supplementary fig. S2B, and Supplementary Table S5). Among the top 20 DMPs, two CpGs (cg22918741 and cg22108941) showed significant correlations with gaze fixation time on the eyes, an index of social cognitive function [17, 18, 28] (Fig. 2C). GAMuT analysis identified 15 significant genes (Supplementary fig. S3), including the lipid phosphate-related protein type 4 (*LPPR4*) gene, which showed a significant association with group status (*P* = 2.90E-05) (Supplementary fig. S4). Notably, the top-ranked DMP, cg18998253, was located within the *LPPR4* gene and was strongly associated with NG as well as group status (see Supplementary Results and Supplementary Tables S5–S7).

### Adolescent brain imaging cohort (Methylation:CM:N = 61, TD:n = 62; MRI:CM:N = 83, TD:N = 154)

Two significant DMPs (cg14209346, cg05646433) were identified after correcting for inflation (Fig. 1, Supplementary Fig. S2C, and Supplementary Table S8). GAMuT analysis identified five significant genes (Supplementary fig. S5 and Supplementary Table S9, and 10), including Armadillo Repeat Containing 5 (*ARMC5*), which showed significant associations with group status and composite maltreatment measures (Supplementary fig. S6). Whole-brain VBM analysis revealed significantly larger GMV in the right orbitofrontal cortex (R.OFrC) and left middle/posterior cingulate gyrus (L.MPCG), and significantly smaller GMV in the left occipital fusiform gyrus (L.OFuG) in the CM group compared to the TD group (Fig. 2D). Among the top 20 DMPs, 13 CpGs were significantly associated with GMV in the R.OFrC, three CpGs with GMV in the L.MPCG, and 12 CpGs with GMV in the L.OFuG (Fig. 2E, and F). Notably, cg04794662, located within the forkhead box protein 1 (*FOXP1*) gene and ranked among the top 20 DMPs, exhibited significant associations with GMV across all three identified brain regions (see Supplementary fig. S7, Supplementary Results and Supplementary Tables S8).

### Meta-analysis

**Fig. 3:**
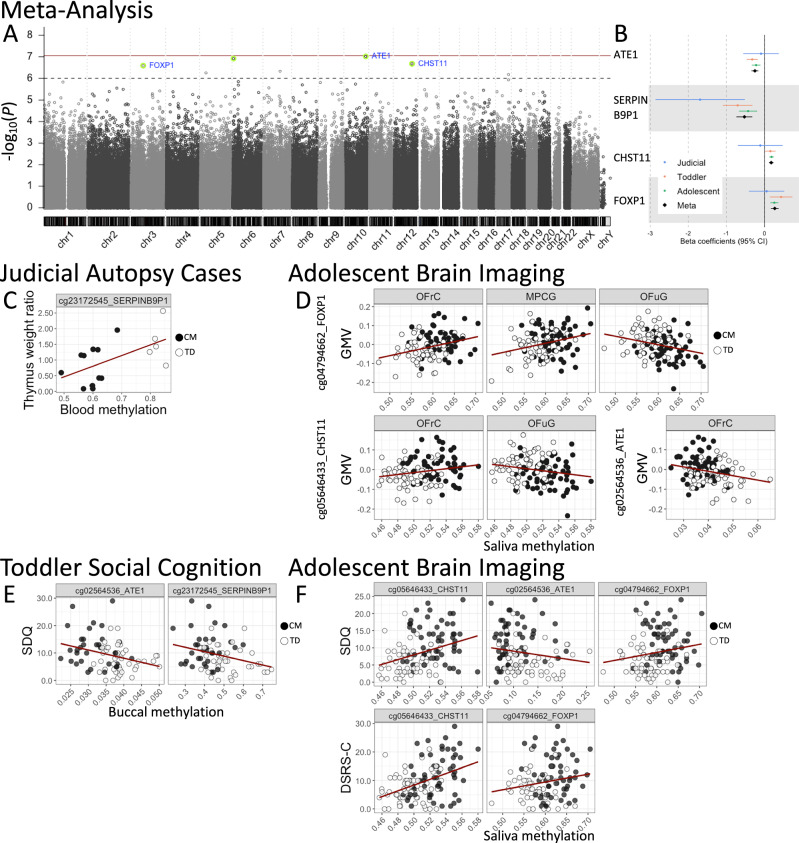
Four significant CpGs identified by epigenome-wide meta-analysis and their associations with biological endophenotypes and clinical outcomes across cohorts. **Meta-Analysis:** (**A**) Manhattan plot (*N* = 226). The horizontal solid line (dark red) indicates *P* = 9.0E-08, and the dashed line (gray) indicates *P* = 1.0E-06. The four probes that reached *q*-value significance are highlighted in light green and labeled with their corresponding gene names. For details on the overall quality of the genome-wide analysis—including Q-Q plots and genomic inflation factors—see Supplementary fig. S2. **B** Forest plot for the four significant probes. Error bars represent the SEM. **Judicial Autopsy Cases:** (**C**) Correlation plot for cg23172545_*SERPINB9P1* showing its relationship to the thymus weight ratio (*r* = 0.57, *P* = 0.02). **Adolescent Brain Imaging:** (**D**) Correlation plots for cg04794662_*FOXP1* (R.OFrC: *r* = 0.31, *P* = 5.4E-04; L.MPCG: *r* = 0.27, *P* = 0.003; L.OFuG: *r* = −0.27, *P* = 0.003), cg05646433_*CHST11* (R.OFrC: *r* = 0.20, *P* = 0.03; L.OFuG: *r* = −0.20, *P* = 0.03) and cg02564536_*ATE1* (R.OFrC: *r* = −0.24, *P* = 0.008) with each of the GMV measures in these regions. Refer to Supplementary Table S11 for detailed statistical analyses. **Toddler Social Cognition and Adolescent Brain Imaging:** (**E**, **F)** Correlation plots illustrating associations between methylation levels and clinical measures (SDQ and DSRS-C). Toddler cohort: cg02564536_*ATE1* (SDQ: *r* = −0.31, *P* = 0.007) and cg23172545_*SERPINB9P1* (SDQ: *r* = −0.29, *P* = 0.011) showed significant correlations. Adolescent cohort: cg05646433_*CHST11* correlated significantly with both SDQ (*r* = 0.32, *P* = 0.0003) and DSRS-C (*r* = 0.42, *P* = 3.0E-06). Additionally, cg02564536_*ATE1* showed a significant negative correlation with SDQ (*r* = −0.26, *P* = 0.0045), and cg04794662_*FOXP1* showed marginally significant positive correlations with SDQ (*r* = 0.17, *P* = 0.058) and DSRS-C (*r* = 0.18, *P* = 0.053).

Four significant CpGs were identified in the meta-analysis (Fig. 3A, and Supplementary Table S11 ; the effect of sex differences is shown in Supplementary Table S12). These CpGs were located within the arginyltransferase 1 (*ATE1*) (Supplementary fig. S8), serine protease inhibitor family B member 9 pseudogene 1 (*SERPINB9P1*, a long non-coding RNA gene), carbohydrate sulfotransferase 11 (*CHST11*) (Supplementary fig. S9), and forkhead box protein 1 (*FOXP1*) (Supplementary fig. S10) genes. Among these CpGs, cg02564536 (*ATE1*) and cg23172545 (*SERPINB9P1*) showed lower methylation levels in the CM group compared to the TD group, whereas cg05646433 (*CHST11*) and cg04794662 (*FOXP1*) exhibited higher methylation levels in the CM group (Fig. 3B). Notably, cg05646433 and cg04794662 were also identified among the top 20 DMPs in the Adolescent Brain Imaging cohort. Three CpGs (*ATE1*, *CHST11*, *FOXP1*) showed low heterogeneity (*I²* ≤ 2.06%), indicating stable effects across cohorts. Although *SERPINB9P1* exhibited moderate heterogeneity (*I²* = 58.98%), the Q-statistic was not statistically significant (*P* = 0.075), suggesting limited statistical evidence for heterogeneity.

To further investigate associations between these four CpGs and relevant endophenotypes, we conducted additional analyses within each cohort. In the Judicial Autopsy Cases, cg23172545 (*SERPINB9P1*) correlated significantly with thymus weight ratio (Fig. 3C). In the Toddler cohort, none of the four CpGs showed significant associations with gaze fixation time on the eyes. In the Adolescent cohort, cg04794662 (*FOXP1*) was significantly associated with GMV in all three brain regions identified by VBM; cg05646433 (*CHST11*) correlated significantly with GMV in the R.OFrC and L.OFuG; and cg02564536 (*ATE1*) negatively correlated with GMV in the R.OFrC (Fig. 3D). Associations with clinical measures (SDQ and DSRS-C) revealed that, in the Toddler cohort, cg02564536 (*ATE1*) and cg23172545 (*SERPINB9P1*) negatively correlated with SDQ scores (Fig. 3E, Supplementary Table S11). In the Adolescent cohort, cg05646433 (*CHST11*) methylation positively correlated with both SDQ and DSRS-C scores, cg02564536 (*ATE1*) negatively correlated with SDQ scores, and cg04794662 (*FOXP1*) showed marginally significant positive correlations with both measures (Fig. 3F, Supplementary Table S11). Detailed results are described in the Supplementary Results and Supplementary Table S11.

### Maltreatment history

**Fig. 4:**
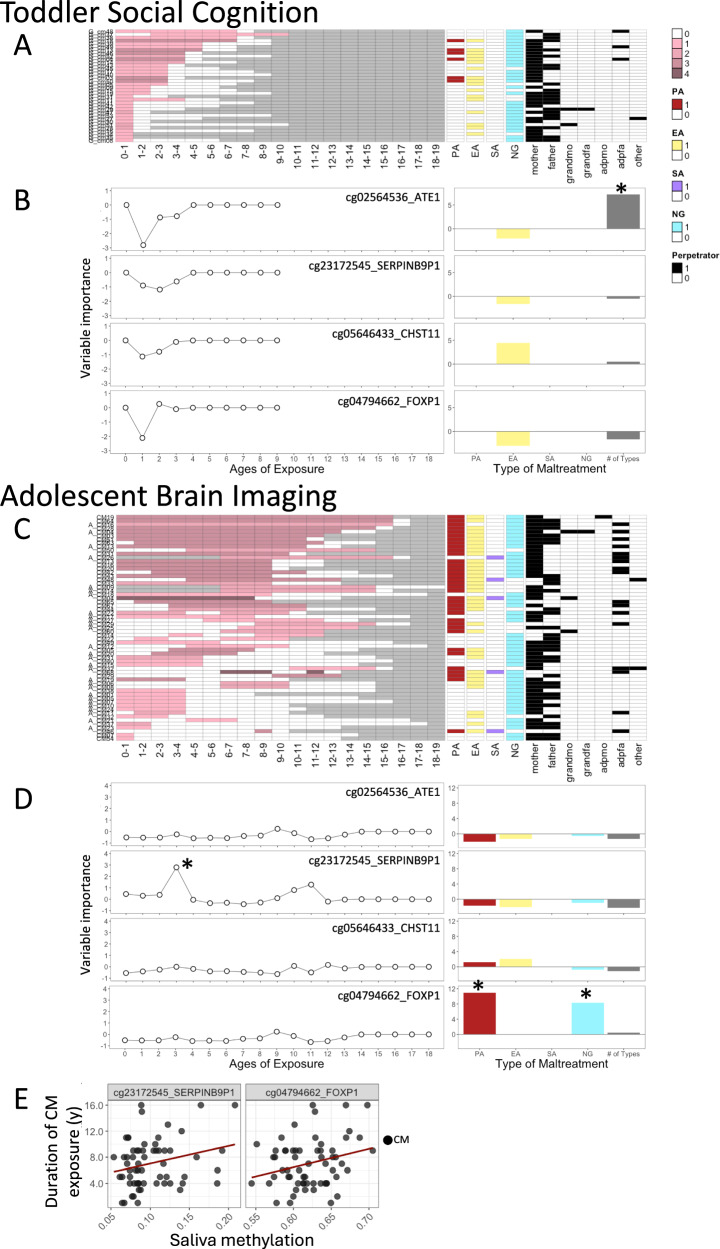
Four significant CpGs identified by epigenome-wide meta-analysis and their associations with timing, type, and duration of maltreatment exposure in the Toddler Social Cognition and Adolescent Brain Imaging cohorts. **Toddler Social Cognition and Adolescent Brain Imaging:** (**A**) and (**C**) Structure of maltreatment history for each individual. Five-step gradation (pink blocks) indicates the number of CM types each individual was exposed to at each age. Gray blocks indicate ages not yet reached by the individual. PA: physical abuse; EA: emotional abuse; SA: sexual abuse; NG: neglect; mother: biological mother; father: biological father; grandmo: grandmother; grandfa: grandfather; adpmo: adoptive mother; adpfa: adoptive father. **Toddler Social Cognition and Adolescent Brain Imaging:** (**B**) and (**D**) Sensitive-period analyses assessing maximal sensitivity by age of exposure (independent of maltreatment type/number) and by maltreatment type/number (independent of age of exposure). Random forest regression with conditional trees was used to evaluate the relative importance of maltreatment exposures during specific age windows (0–18 years) or specific maltreatment types/numbers for methylation at each of the four CpG sites. Relative importance was indicated by the degradation in model fit (increase in mean squared error, MSE) when each factor was permuted. *P < 0.05. **Adolescent Brain Imaging:** (**E**) Correlation plots illustrating significant associations between duration of maltreatment exposure and methylation levels at cg23172545_*SERPINB9P1* and cg04794662_*FOXP1*.

Sensitive-period analyses were performed to investigate the age at which maltreatment exposure significantly contributed to methylation at each CpG site (Fig. 4A, B, C, and D). In the Adolescent cohort, maltreatment exposure at age three significantly contributed to methylation at cg23172545 (*SERPINB9P1*) (*r* = 0.42, *P* = 0.03; Fig. 4D). Additionally, analyses examining the type of maltreatment contributing to methylation revealed that, in the Toddler cohort, the number of maltreatment types experienced significantly contributed to methylation at cg02564536 (*ATE1*) (*r* = 0.30, *P* = 0.049; Fig. 4B). In the Adolescent cohort, PA (*P* = 0.02) and NG (*P* = 0.02) significantly contributed to methylation at cg04794662 (*FOXP1*) (*r* = 0.38; Fig. 4D). Furthermore, methylation levels at cg23172545 (*SERPINB9P1*:*r* = 0.25, *P* = 0.048) and cg04794662 (*FOXP1*:*r* = 0.27, *P* = 0.04) were significantly correlated with the duration of maltreatment exposure, indicating higher methylation with longer exposure periods (Fig. 4E, Supplementary Table S13). No other combinations showed significant associations.

### Enrichment analysis

GO enrichment analysis identified 167 significant biological process (BP) terms (*P* < 0.05) enriched among the top 1500 CpGs from the meta-analysis (Supplementary Table S14). These 167 BP terms were visualized using a GO semantic similarity matrix [34], resulting in 11 functionally enriched clusters (Supplementary fig. S11). KEGG pathway enrichment analysis revealed 15 significant pathways (*P* < 0.05; Supplementary Table S15). An integrated overview of these enrichment results highlighted genes involved in glutamate receptor-mediated synaptic signaling modulated by acetylcholine.

### Evaluation of candidate CpGs from previously published studies

We evaluated candidate CpGs previously reported in five independent studies [8, 10–13] using the analytical framework established in our meta-analysis (see Supplementary Results, Supplementary Table S16).

### MRS in the validation dataset (GSE118940)

**Fig. 5:**
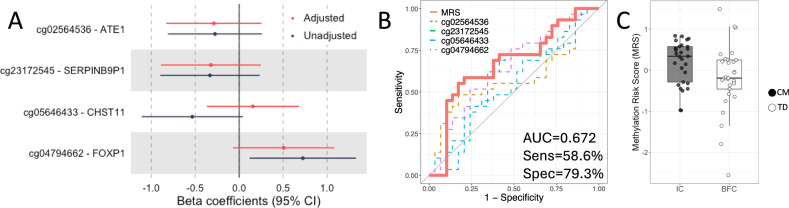
Receiver Operating Characteristic (ROC) curves for logistic regression models incorporating the Methylation Risk Scores (MRS) in an independent public dataset (GSE118940). **A** The coefficient estimates obtained from logistic regression models (with and without adjustment for age, sex, and blood cell proportions) for the four probes that constituting the MRS. **B** ROC curves illustrating the classification performance of these logistic regression models. AUC: area under the curve; Sens: sensitivity; Spec: specificity. **C** Box plot presenting the adjusted-model MRS values in the institutional care (IC) and the biological family care (BFC) group. Error bars indicate the standard error of the mean (SEM).

Coefficient estimates for four CpG sites from adjusted and unadjusted models are presented in Fig. 5A. Because the directionality of associations for all CpG sites was consistent in the adjusted model, we adopted this model for subsequent analyses. Individual MRS values ranged from −2.56–1.49 in this dataset. Optimal cutoff values for group classification were determined using receiver operating characteristic (ROC) curve analysis, resulting in thresholds of ≥0.265 (AUC = 0.672; Fig. 5B). A significant difference in MRS was observed between the institutional care (IC) and biological family care (BFC) groups (*t* = 2.1, *P* = 0.04; Fig. 5C). Taken together, these findings suggest that, even after accounting for social background, higher MRS values are associated with greater exposure to adverse early-life experiences.

## Discussion

We identified CM-associated methylation differences specific to each cohort and analyzed their associations with relevant biological indicators. Subsequently, a meta-analysis integrating these results identified four significant CpGs located within the *ATE1*, *SERPINB9P1*, *CHST11*, and *FOXP1* genes. Methylation differences in these genes were also associated with relevant biological indicators, clinical measures, and maltreatment history, suggesting that these epigenetic alterations may contribute to neurobiological changes and the emergence of psychosocial difficulties commonly observed in maltreated children. Detailed discussions of cohort-specific findings and clinical implications are provided in the Supplementary Discussion.

Of particular interest are findings related to *FOXP1*. *FOXP1* is a tumor suppressor gene and, along with its homolog *FOXP2*, plays an essential role in brain development [37]. In contrast to *FOXP2*, which is primarily associated with language disabilities, *FOXP1* has been linked to a neurodevelopmental disorder known as *FOXP1* syndrome, characterized by autism spectrum disorder (ASD)-like symptoms and intellectual disabilities [37]. *FOXP1* syndrome typically arises from genetic mutations or deletions in the *FOXP1* gene, resulting in a significant reduction or complete loss of *FOXP1* expression in the brain. This loss-of-function severely disrupts normal *FOXP1*-dependent neurodevelopmental processes, leading to the observed clinical phenotype. Although the methylation changes identified in our study are unlikely to cause such extreme reductions in *FOXP1* expression as seen in genetic mutations or deletions, increased methylation at this CpG site (cg04794662) has been reported to be modestly but significantly associated with decreased *FOXP1* expression levels in children’s blood (log_2_FC = −0.02, *P* < 0.05) [38]. To interpret the biological relevance of peripheral methylation findings, we utilized our brain-peripheral tissue methylation correlation database (AMAZE-CpG), which provides correlations between methylation levels in brain and peripheral tissues. The AMAZE-CpG results indicated that methylation at this CpG site tends to be positively correlated between brain and peripheral tissues (brain-blood:*rho_adj* = 0.40, *P_adj* = 0.09; brain-saliva:*rho_adj* = 0.43, *P_adj* = 0.07) [36]. Thus, although caution is warranted when interpreting peripheral methylation findings, it may be reasonable to discuss potential epigenetic effects on the brain, given that peripheral methylation at this site appears to exhibit similar dynamics to brain methylation and correlates with brain structural features uniquely associated with CM.

Methylation of *FOXP1* was significantly associated with all three atypical brain structures identified in the CM group specifically larger GMVs in the R.OFrC and L.MPCG, and smaller GMV in the L.OFuG. Interestingly, each of these structural differences has also been reported in children with ASD [39, 40]. Furthermore, the directionality of these structural differences aligns with findings from a recent large-scale mega-analysis conducted by the ENIGMA consortium comparing ASD (*N* = 1655–1657) and Control (*N* = 1568–1572) groups at an average age of 15 years (Cohen’s *d* with *Ps* < 0.002:medial orbitofrontal = 0.15, posterior (parietal) cingulate = 0.13, fusiform = −0.19) [41]. Although our study did not directly examine ASD, the observed similarity in structural brain characteristics between CM and ASD suggests that maltreatment exposure may influence *FOXP1*-related neurodevelopmental processes, potentially contributing to ASD-like neurodevelopmental symptoms. Additionally, data from the Brainspan database confirm that these brain regions overlap with areas of *FOXP1* expression (Supplementary fig. S12). Given that *FOXP1* syndrome involves severe disruptions in *FOXP1*-dependent neurodevelopmental pathways, milder epigenetic alterations in *FOXP1* induced by CM may partially overlap with these pathways, resulting in similar structural brain changes. Regarding maltreatment history, no clear sensitive period was identified; however, significant associations were observed for PA and NG. Given the severe and chronic nature of both PA and NG [1], it is possible that these more intense or prolonged forms of maltreatment are particularly associated with epigenetic alterations in *FOXP1*. PA involves direct physical harm, while NG represents chronic deprivation of essential emotional and physical care; both experiences may profoundly disrupt neurodevelopmental processes regulated by *FOXP1*. Clinically, maltreated children often exhibit symptoms resembling ASD and attention-deficit/hyperactivity disorder (ADHD) [42, 43]. Therefore, differentiating between maltreated children and those with neurodevelopmental disorders can sometimes be difficult. These neurodevelopmental-like symptoms may thus arise, at least in part, from epigenetic abnormalities involving altered DNA methylation of the *FOXP1* gene.

The lower methylation level in the *ATE1* gene was associated with larger GMV in the R.OFrC, a region responsible for emotional regulation [44]. During adolescence, cortical structures typically become more refined and sophisticated, resulting in smaller regional GMVs [45]. Thus, the observation that the R.OFrC remained larger in the CM group may reflect relative developmental immaturity compared to the TD group. Regarding maltreatment history, the number of maltreatment types significantly predicted methylation levels in toddlers; however, no clear sensitive period was identified. The *ATE1* gene catalyzes protein arginylation, a process involved in multiple biological functions, including cardiovascular development, angiogenesis, and neural development [46]. A large genome-wide association study (GWAS) examining interactions between genetic polymorphisms and childhood environment in relation to alcohol addiction identified an SNP (rs4752622) in *ATE1* that interacted with childhood experiences of “felt loved” and was protectively associated with alcohol drinking frequency, suggesting a link to childhood attachment [47]. Additionally, *ATE1* is crucial for normal brain development in mice, and its absence results in abnormal neurite outgrowth and mislocalization of doublecortin in growth cones [48]. Our AMAZE-CpG database indicated a significant positive correlation between brain and blood methylation at this CpG site (*rho_adj* = 0.50, *P_adj* = 0.03) [36]. Taken together, these findings suggest that altered methylation of *ATE1* may be associated with delayed R.OFrC development, potentially reflecting disrupted attachment formation related to CM exposure.

The higher methylation level in the *CHST11* gene was associated with larger GMV in the R.OFrC and smaller GMV in the L.OFuG. *CHST11* belongs to the glycosulfotransferase family, which modifies glycosylation scaffolds involved in extracellular signaling and adhesion mechanisms [49]. In the brain, *CHST11* contributes to the production of chondroitin-4-sulfate, a critical component of the extracellular matrix that regulates neural plasticity, social memory, and anxiety [50]. Previous research has reported that another CpG site (cg04130728) within *CHST11* exhibited higher methylation in veterans with PTSD compared to controls, in both blood and brain tissues [51]. Our findings similarly indicated higher methylation levels in the CM group compared to the TD group, consistent with this prior evidence. Additionally, our AMAZE-CpG database showed a positive correlation trend between brain and buccal methylation at this CpG site (*rho_adj* = 0.44, *P_adj* = 0.06) [36]. Collectively, these observations suggest that *CHST11* methylation may be associated with traumatic experiences. Indeed, methylation at this site correlated significantly with depressive symptoms (DSRS-C; *r* = 0.37, *P* = 4.1E–05) and showed a trend-level correlation with behavioral and social difficulties (SDQ; *r* = 0.17, *P* = 0.06). Given that depressive symptoms frequently co-occur with PTSD [52], these findings are consistent with a trauma-related mechanism.

Methylation of *SERPINB9P1* (cg23172545) was associated with lower thymus weight ratios but was not significantly related to brain GMVs. Regarding maltreatment history, a sensitive period was observed at age three in adolescents, although no specific maltreatment types were identified. *SERPINB9P1* has previously been reported to regulate osteogenic differentiation of bone marrow stromal cells [53]. Considering these biological functions, methylation at this CpG site may be associated with impaired immune function and reduced growth rates observed in maltreated children. The identification of a sensitive period at age three is particularly noteworthy, as this developmental stage corresponds to a period of rapid growth, raising the possibility that CM exposure during this critical window could have lasting physiological consequences.

The results of the GO and KEGG enrichment analyses, conducted to identify biological functions enriched among the top-ranked genes from our meta-analysis, revealed that “cholinergic” and “synaptic transmission” pathways were commonly enriched in both analyses, while “glutamatergic synapse” was specifically enriched in the KEGG analysis. Animal studies have demonstrated that synaptic transmission mediated by AMPA-type glutamate receptors plays a critical role in the formation of traumatic memories, a process modulated by acetylcholine secretion [54, 55]. Thus, our findings suggest that the top-ranked genes identified in this meta-analysis may include those involved in synaptic transmission and related molecular mechanisms potentially underlying the encoding and retrieval of traumatic memories. These epigenetic modifications may reflect molecular adaptations associated with exposure to CM.

Finally, we validated the classification model based on the MRS, constructed from the four CpGs identified in our meta-analysis. Although the publicly available validation dataset differed slightly in its definition of CM [10], the MRS model successfully discriminated between groups with up to 67.2% accuracy. Among the four CpGs, *FOXP1* methylation contributed most significantly to classification accuracy, with effect directionality consistent with our original findings. This underscores the potential utility of *FOXP1* methylation as a biomarker.

This study has several limitations. First, the sample size was relatively small for a genome-wide analysis. Judicial Autopsy Cases are rare, and our inclusion criteria for the Toddler Social Cognition and Adolescent Brain Imaging cohorts required participants who had undergone actual social interventions, further limiting the available sample. Additionally, practical constraints associated with experiments such as brain MRI restricted participant numbers. Thus, our study represents a rigorous, small-scale investigation rather than a permissive, large-scale analysis. Second, nutritional deficiencies, such as famine, have been reported to leave residual effects on the epigenome [56], and Judicial Autopsy Cases may have been particularly affected by such factors. Although maltreated children in the other two cohorts did not exhibit nutritional problems at the time of participation, past nutritional deficiencies may have coincided with their exposure to CM. The effects of CM inherently involve multiple variables, including nutritional, physical, and psychological factors. Future studies that separately analyze these factors may further elucidate the underlying mechanisms. Third, our findings revealed significant differences in estimated cell-type proportions between CM and TD groups (Supplementary Table S17), consistent with previous reports linking CM to immune alterations [57, 58]. However, differences in cell proportions alone do not directly indicate impaired immune function or chronic inflammation. Further studies directly assessing immune function (e.g., cytokine profiles, immune response assays) are needed to clarify the functional implications of these findings.

In conclusion, our study identified novel CM-associated methylation signatures through a multi-cohort epigenome-wide analysis and further revealed common methylation sites via meta-analysis. These findings enhance our understanding of epigenetic mechanisms underlying adverse outcomes associated with CM. Ultimately, this research may facilitate therapeutic strategies aimed at reversing epigenetic changes, support biomarker development for prevention and psychotherapy, and represent an important step toward breaking the intergenerational cycle of CM.

## Supplementary information

Supplementary Information

Supplementary Tables

Supplementary Tables S4

Supplementary Figures

## Data Availability

The main statistical data supporting the findings of this study are provided in Supplementary Tables. The DNA methylation microarray data generated in this study have been deposited in the Gene Expression Omnibus (GEO) database under the SuperSeries accession number GSE239522 (SubSeries:GSE239517, GSE239518, GSE239520, and GSE239521). For additional information or inquiries regarding data access, please contact the corresponding authors.

## Code availability

All analyses were performed using previously published and publicly available codes, such as PGC-pipeline QC and meta-analysis, GAMuT, and sensitive-period analyses (Supplementary Methods).
