# Supplementary material for: Multi-epigenome-wide analyses and meta-analysis of child maltreatment in judicial autopsies and intervened children and adolescents
Source: Mol Psychiatry. 2025 Sep 16;31(3):1253–64. doi: 10.1038/s41380-025-03236-1 (PMC12916479; doi:10.1038/s41380-025-03236-1)
Supplement: Supplementary file 1 — Supplementary Information [file 41380_2025_3236_MOESM1_ESM.pdf]

Supplementary Information

Supplementary Methods

Quality control (QC) procedures for blood, buccal, and saliva DNA methylation

Five hundred nanograms of DNA were bisulfite-converted using the EZ DNA Methylation™ Kit (Zymo Research, D5002). Genome-wide DNA methylation was assessed using the Infinium HumanMethylationEPIC BeadChip Kit (WG-317-1002; Illumina). Samples were grouped by individual and balanced across chips to minimize batch-related confounding effects [1]. Arrays were scanned using the Illumina iScan platform. Methylation levels (β-values) were calculated using the minfi R package [2], and the standardized QC pipeline from the Psychiatric Genomics Consortium–Epigenome-Wide Association Studies [3, 4] was applied separately to each cohort. Using CpGassoc [5], we excluded samples with probe detection call rates <90% and samples with average intensity values either <50% of the experiment-wide sample mean or <2,000 arbitrary units (AU). Low-quality probes (detection *P*-values >0.01) were set as missing, and probes missing in >10% of samples were removed. Probes containing single nucleotide polymorphisms (SNPs; based on the 1000 Genomes Project) within 10 base pairs of the target CpG were retained, while cross-hybridizing probes were excluded. After QC, a total of 820,440 (Judicial Autopsy Cases), 819,680 (Toddler Social Cognition), and 816,791 (Adolescent Brain Imaging) probes remained for analysis. Single-sample Noob (ssNoob) normalization was performed using minfi. To correct for chip and positional batch effects, ComBat (sva) [6] was applied, protecting group, age, and sex. This approach was chosen because singular value decomposition analysis using ChAMP [7] revealed residual confounding effects from unwanted non-biological variables, despite the balanced experimental design (Supplementary Figure S1). Group, age, and sex were thus preserved from ComBat correction for subsequent genome-wide analyses. Logit-transformed β-values (M-values) were used for statistical analyses. Cellular heterogeneity for each sample was estimated using the Robust Partial Correlation method implemented in EpiDISH [8].

Quality control (QC) procedures for brain DNA methylation

Two hundred fifty nanograms of DNA were bisulfite-converted using the EZ DNA Methylation™ Kit (Zymo Research, D5002) and restored using the Illumina HD FFPE Restoration Kit (Illumina). Microarray analysis was performed as described above. However, due to generally poor and highly variable data quality across samples, the standard QC criteria described previously could not be uniformly applied. Therefore, we implemented customized QC criteria, retaining only probes for which at least three individuals per group had detection *P*-values < 0.05.

Autopsied thymus weight ratio calculation

Thymus weight is a forensic index reflecting the severity and duration of child abuse or neglect [9]. Because thymus weight increases with age and developmental stage, we calculated a thymus weight ratio relative to the lower limit of the normal range for each age group. The normal range for thymus weight by age was based on criteria established by the Medico-Legal Society of Japan [10]. Specifically, the lower limit was defined as the mean thymus weight minus one standard deviation for each age group. The thymus weight ratio was then calculated by dividing the observed thymus weight by this lower limit (Supplementary Table S1). A group comparison

was performed using a *t*-test. Pearson's correlation analyses were conducted to examine associations between thymus weight ratios and methylation levels identified in the genome-wide analyses. For correlation analyses, methylation values were adjusted by regressing out covariates included in the genome-wide analyses. Statistical significance was set at $P < 0.05$.

Assessment of social cognitive function by gaze patterns for Toddler Social Cognition cohort

Participants' gaze patterns were measured using Gazefinder® (JVC KENWOOD Corporation, Kanagawa, Japan), an eye-tracking system designed to record responses to visual stimuli. Gaze fixation time on the eye region during facial image presentation was used as an index of social cognitive function, based on previous findings that children with CM and autism spectrum disorder (ASD) spend significantly less time fixating on the eyes compared to typically developing (TD) children [11-13]. Detailed experimental procedures are described in these prior studies. Pearson's correlation analyses were conducted to examine associations between gaze fixation time on the eyes and methylation levels identified in the genome-wide analyses. For correlation analyses, methylation values were adjusted by regressing out covariates included in the genome-wide analyses. Statistical significance was set at $P < 0.05$.

Brain image acquisition and preprocessing

Structural MRI data were acquired from 237 participants. Imaging for 137 participants was performed using a GE Discovery MR750 3-Tesla scanner equipped with a 32-channel head coil (GE Healthcare, Milwaukee, WI, USA), while imaging for the remaining 100 participants was conducted using a GE Signa PET/MR 3-Tesla scanner with an 8-channel head coil (GE Healthcare, Milwaukee, WI, USA). T1-weighted anatomical images were obtained using a fast-spoiled gradient recalled imaging sequence with the following parameters: for 137 participants, voxel size = 1 × 1 × 1 mm, TE = 1.99 ms, TR = 6.38 ms, flip angle = 11°; for 100 participants, voxel size = 1 × 1 × 1 mm, TE = 3.24 ms, TR = 8.46 ms, flip angle = 11°.

Structural imaging data were preprocessed using Voxel-Based Morphometry (VBM) implemented in Statistical Parametric Mapping (SPM12; Wellcome Trust Centre for Neuroimaging, London, UK) running on MATLAB R2021b (MathWorks, Natick, MA, USA). T1-weighted images were segmented into gray matter (GM), white matter (WM), cerebrospinal fluid, and skull/scalp compartments using tissue probability maps. The Diffeomorphic Anatomical Registration through Exponentiated Lie Algebra (DARTEL) algorithm was applied to segmented brain tissues to generate a study-specific template, enabling accurate inter-subject registration and improved alignment of smaller internal structures [14]. Segmented GM images were spatially normalized to an isotropic voxel resolution of 1.5 mm. Volume changes induced by normalization were corrected using modulation. Finally, spatially normalized GM images were smoothed using a Gaussian kernel with a full width at half maximum (FWHM) of 10 mm.

Brain Voxel-Based Morphometry (VBM)

Regional differences in gray matter volume (GMV) between groups were analyzed using multiple regression analyses implemented in SPM12. Potential confounding variables, including age, full-scale IQ (FSIQ), scanner type, and total GMV, were modeled, and their variance was removed from subsequent analyses. Total GMV was calculated from segmented GM images using the "TissueVolumes" utility in the SPM12 batching system. A gray matter majority optimal

threshold mask, created based on a study-specific sample, was applied to exclude non-GM voxels from the analyses [15]. The resulting voxel-wise comparisons generated statistical parametric maps of *t*-statistics (SPM[*t*]), which were subsequently transformed into unit-normal distributions (SPM[*Z*]). Significant clusters were anatomically localized using the Neuromorphometric Atlas (Neuromorphometrics Inc.). To correct for multiple comparisons across the entire brain, statistical significance was set at a voxel-level threshold of $P < 0.001$ (uncorrected) for height, with cluster-level correction at $P < 0.05$.

Pearson's correlation analyses were conducted to examine associations between GMV and methylation levels identified in genome-wide analyses. For these correlations, methylation values were adjusted by regressing out covariates included in the genome-wide analyses. Correlation analyses utilized residual GMV values obtained from group comparisons after adjusting for control variables. Adjusted eigenvariates, representing linearly transformed estimates of GMV, were used in these correlation analyses. Statistical significance for correlation analyses was set at $P < 0.05$.

Maltreatment history

The presence of a potential "sensitive period," during which exposure to maltreatment may have a stronger association with methylation alterations, was assessed using random forest regression with conditional inference trees (cforest), as described in our previous studies [16]. Two separate analyses were conducted to evaluate the importance of potential predictors of methylation alterations.

In the first analysis, we examined the relative importance of maltreatment exposure during specific developmental periods. Exposure to any form of maltreatment—physical abuse (PA), emotional abuse (EA), sexual abuse (SA), or neglect (NG)—was scored annually from birth to 18 years of age, with each year coded as either zero (no exposure) or one (exposure). In the second analysis, we evaluated the comparative importance of exposure to different maltreatment types (PA, EA, SA, and NG). Additionally, the total number of overlapping maltreatment types experienced was included as a predictor, as multiplicity of exposure may represent a more critical determinant of methylation alterations than any single type of maltreatment alone. Each random forest consisted of 200 trees, with four variables randomly selected for evaluation at each node.

Additionally, Pearson correlation analyses were conducted to examine associations between the duration of maltreatment exposure (based on documented maltreatment histories) and methylation levels.

Validation analysis

To validate the CpGs identified in the meta-analysis, we applied the methylation risk score (MRS) [17] derived from our meta-analysis to an independent validation dataset (GSE118940). This dataset comprised blood DNA methylation data measured by the Illumina Methylation EPIC array from 29 children residing in orphanages (institutional care group, IC) and 29 children raised by their biological families (biological family care group, BFC), aged 8–35 months [18]. Data preprocessing involved QC procedures using CpGassoc [5], similar to those applied in the present study, including the removal of cross-hybridizing probes. However, normalization was performed using the Beta-Mixture Quantile (BMIQ) method [19]. Individual-level data on age and

sex were publicly available; however, for one participant whose chronological age was missing, methylation age estimated using Horvath's epigenetic clock [20] was substituted. Blood cell-type proportions were estimated using EpiDISH [8]. Logistic regression analyses were conducted using normalized β-values adjusted by regressing out the effects of age, sex, and proportions of five blood cell types (excluding neutrophils).

Supplementary Results

Detailed results for demographic characteristics of participants

IQ/DQ scores were lower in the CM group compared to the TD group, representing a potential confounding factor; however, lower IQ/DQ is commonly observed among maltreated children. Although many previous studies have either not assessed IQ or have not adjusted for it in their analyses [18, 21-23], we included IQ/DQ as a covariate in our genome-wide analyses for the Toddler Social Cognition and Adolescent Brain Imaging cohorts to ensure accuracy.

Detailed results for Judicial Autopsy Cases

After applying customized QC criteria (requiring at least three individuals per group with detection *P*-values < 0.05), 567,433 probes and 23,244 genes remained available for brain methylation analyses. However, due to varying sample sizes across probes, genome-wide analyses were not feasible. Therefore, we compiled a comprehensive statistical summary of brain methylation results for individual probes (Supplementary Table S4).
Brain-blood DNA methylation correlations for these top 20 DMPs are summarized in Supplementary Table S3. Additionally, detailed statistical information for the seven CpGs significantly correlated with thymus weight ratio is provided in Supplementary Table S3.

Detailed results for Toddler Social Cognition cohort

Brain-buccal DNA methylation correlations for the top 20 DMPs are summarized in Supplementary Table S5. GAMuT analysis identified 15 significant genes (Supplementary Figure S3 and Supplementary Table S6). Secondary analyses revealed that four CpGs within the *LPPR4* gene were significantly associated with specific maltreatment indicators (Supplementary Figure S4, Supplementary Table S7). Associations between the top 20 DMPs and SDQ scores are also presented in Supplementary Table S5.

Detailed results for Adolescent Brain Imaging cohort

Brain-saliva DNA methylation correlations for the top 20 DMPs are summarized in Supplementary Table S8. GAMuT analysis identified five significant genes (Supplementary Figure S5 and Supplementary Table S9,10). Among these, the Armadillo Repeat Containing 5 (*ARMC5*) gene, comprising 33 CpGs, showed significant associations with group status (*P* = 6.31E-06) and composite maltreatment measures (*P* = 4.46E-05) (Supplementary Figure S6). Secondary analyses revealed that two CpGs within *ARMC5* were significantly associated with specific maltreatment indicators (Supplementary Table S10). Specifically, cg08509907, which was among the top 20 DMPs (Supplementary Table S8) and associated with GMV in the R.OFrC and L.OFuG, and cg10451425 were strongly associated with EA (*P* = 1.4E-04 and *P* = 6.8E-05, respectively) and NG (*P* = 1.2E-04 and *P* = 0.002, respectively), in addition to their associations with group status (*P* = 7.79E-07 and *P* = 3.35E-05, respectively) (Supplementary Table S10).

Associations between the top 20 DMPs and scores on the SDQ and DSRS-C are presented in Supplementary Table S8.

Detailed results for Meta-analysis

To explore potential sex differences, we tested interactions between group and sex for these four CpGs separately in each cohort (Supplementary Table S12). A significant interaction was observed only for cg02564536 (*ATE1*) in the Adolescent cohort (interaction $P = 0.003$), driven primarily by a large effect size in males (Cohen's $d = -1.38$, 95% CI: -1.89 to -0.87), whereas the effect in females was small and non-significant (Cohen's $d = -0.22$, 95% CI: -0.81 to 0.37). No significant interactions were found in the Judicial Autopsy or Toddler cohorts (all $P > 0.05$). Brain-peripheral tissue DNA methylation correlations for the four significant CpGs (cg02564536, cg23172545, cg05646433, and cg04794662) are summarized in Supplementary Table S11.

Detailed results for evaluation of candidate CpGs from previously published studies

We evaluated candidate CpGs previously reported in five independent studies using the analytical framework established in our meta-analysis (Supplementary Table S14). Of the 2,868 CpGs reported by Yang et al. [24] using the 450K array, 202 (9.8%) of the 2,060 CpGs available on the EPIC array were significant and showed consistent directionality. None of the CpGs reported by Kumsta et al. [25]—including 10 DMPs and nine CpGs comprising a DMR—were significant in our analysis. Of the 550 CpGs reported by Papale et al. [22] using the 450K array, 25 (5.0%) of the 504 CpGs present on the EPIC array were significant and directionally consistent. Among the 156 CpGs reported by Naumova et al. [18], only one (<1%) was significant and consistent in directionality. Finally, of the 15 CpGs reported by Sumner et al. [23] using the EPIC array, one CpG (6.7%) was significant and consistent in directionality.

Supplementary Discussion

Judicial Autopsy Cases

No significant DMPs in blood were identified after correcting for inflation. However, given the extreme severity of adversity experienced by these cases—abuse severe enough to result in death—it is plausible that profound disturbances in the hypothalamic-pituitary-adrenocortical (HPA) axis occurred, potentially manifesting as thymic gland atrophy. Indeed, among the top 20 DMPs identified, seven CpGs showed significant correlations with thymic atrophy, suggesting that these epigenetic alterations may reflect immune system dysfunction. Consistent with this interpretation, epigenetic alterations in immune-related genes have previously been reported in the context of post-traumatic stress disorder (PTSD) [26].

Our study is the first to examine genome-wide DNA methylation changes in the brains of maltreated children, utilizing extremely rare and valuable autopsy cases. Although the limited sample size precluded a comprehensive genome-wide characterization, individual results provided in Supplementary Table S4 may serve as a valuable resource for interpreting future studies. We also examined concordance between brain and blood methylation findings. Among the seven CpGs discussed above, two sites (cg22646780 and cg16523850) exhibited significant group differences in brain methylation. However, since these CpGs are not annotated to specific genes, their functional implications remain unclear at present. Taken together, these findings suggest that epigenetic modifications in immune-related genes may represent a

biological mechanism linking severe child maltreatment to long-term neurobiological and immunological consequences. However, caution is warranted when interpreting these findings, as malnutrition—particularly prevalent among neglected children—can also lead to thymic atrophy [27]. For example, methylation at cg08734093 in the mechanistic target of rapamycin kinase (*MTOR*) gene, known to respond to cellular stressors such as DNA damage and nutrient deprivation[28], was among the highly ranked (87th) CpGs identified in our analysis. Additionally, several cases in this study involved traumatic injuries directly related to the cause of death, suggesting that some of the identified CpGs may reflect acute inflammatory responses triggered by severe physical trauma, such as hemorrhage. Thus, further studies are necessary to distinguish epigenetic changes specifically attributable to maltreatment from those resulting from malnutrition or acute injury-related inflammation.

Toddler Social Cognition cohort

In this cohort, nine significant DMPs were identified. We previously reported that maltreated children in this age group spend less time gazing into the eyes of others[11, 12], a behavioral feature indicative of impaired social functioning. Among the top 20 DMPs identified, two CpGs (cg22918741 and cg22108941) correlated with this behavioral phenotype, suggesting a potential association with reduced sociability. However, as these two CpGs are not annotated to specific genes, their functional implications remain unclear at present.

Notably, the phospholipid phosphatase-related 4 (*LPPR4*) gene including 25 CpGs emerged as significant in the GAMuT test and four CpGs (cg15728779, cg11956761, cg08271203, cg18998253) were particularly strongly associated with the EA and NG domains (*adjP*-values < 0.05) (Supplementary Table S6 and S7). One of the sites (cg18998253) was also the top-ranked DMP regardless of correlations with behavioral features. *LPPR4* encodes the Plasticity-Related Gene-1 (PRG-1) protein, which regulates cortical excitatory transmission in glutamatergic neurons and is primarily expressed at neuronal axonal membranes. PRG-1 is known to play an important role in axonal elongation during neurodevelopment [29]. These epigenetic alterations may therefore contribute to neurodevelopmental mechanisms underlying vulnerability in cognitive domains among children who have primarily experienced EA and NG. However, further studies examining detailed associations with brain structure and function are necessary to clarify these relationships.

Adolescent Brain Imaging cohort

Structural brain imaging findings

Previous brain imaging studies provide important context for interpreting the structural brain differences observed in our results. For example, a PET study of women with a history of SA reported increased blood flow in the MPCG and decreased blood flow in the OFuG during memory retrieval of childhood SA among individuals with PTSD [30]. Consistent evidence indicates that the MPCG is involved in encoding and retrieval of episodic and autobiographical memories [31], while the OFuG is implicated in higher-order visual processing, particularly face and facial identity recognition [32], including familiar faces associated with socio-emotional attachment [33, 34]. Furthermore, emotionally arousing memories activate the MPCG along with the hippocampus during retrieval [35]. Anatomically, the MPCG is strongly connected to the parahippocampal gyrus, a critical component of the hippocampal memory system, and also has

connections with the OFrC [36]. rs-fMRI studies have additionally reported stronger functional connectivity between MPCG and OFrC in individuals with depression [37], and the OFrC itself is widely recognized as a key region involved in emotional regulation [38]. Taken together, these findings suggest that structural atypicalities observed in the R.OFrC, L.MPCG, and L.OFuG among adolescents exposed to maltreatment may reflect altered memory encoding and retrieval processes associated with strong negative emotional experiences, including exposure to negative facial expressions from close caregivers. Such structural alterations may contribute to clinical symptoms commonly observed in maltreated individuals, including PTSD and affective dysfunction.

DNA methylation findings and their associations with the atypical brain structures
In this cohort, two significant DMPs (cg14209346 and cg05646433) were identified. One of these CpGs (cg05646433), located within the *CHST11* gene, is discussed in detail in the main manuscript. The other CpG (cg14209346) is not annotated to any specific gene; therefore, its functional implications remain unclear. To further explore the biological relevance of these epigenetic findings, we conducted imaging epigenetics analyses focusing specifically on the top 20 DMPs in relation to the three regional GMVs identified as structurally atypical in CM. Our results revealed that methylation levels at 13 CpGs correlated positively with GMV in the R.OFrC, three CpGs correlated positively with L.MPCG volume, and 12 CpGs correlated negatively with L.OFuG volume. In addition to *FOXP1* (cg04794662), which is discussed in detail in the main manuscript, methylation at cg08509907 within the *ARMC5* gene was notably correlated with GMV in both the R.OFrC and L.OFuG, and significantly associated with PA, EA, and NG domains in the GAMuT test, showing consistent directionality. Furthermore, methylation at this site correlated significantly with SDQ and DSRS-C scores, suggesting its potential relevance to clinical symptoms. *ARMC5* is a tumor suppressor gene previously linked to bilateral adrenocortical macronodular hyperplasia, a condition characterized by cortisol hypersecretion and Cushing's syndrome [39]. Given that CM represents chronic, severe stress exposure, and considering previous evidence of HPA axis dysregulation and elevated cortisol levels in maltreated children [40, 41], abnormal *ARMC5* methylation may reflect an early epigenetic alteration contributing to HPA axis dysfunction. Our previous studies have reported reduced GMV in visual processing regions among individuals exposed to CM [42, 43], suggesting potential disruptions in visual and socio-emotional processing pathways. Although the L.OFuG primarily supports higher-order visual processing (e.g., face recognition linked to socio-emotional attachment), the observed association between *ARMC5* methylation and reduced L.OFuG volume may reflect broader impairments in these pathways. Additionally, the correlation between *ARMC5* methylation and R.OFrC volume suggests that *ARMC5* methylation may also influence brain regions critical for emotional regulation. Taken together, these findings indicate that *ARMC5* methylation could represent a biological mechanism linking chronic stress from CM to structural brain alterations involved in emotional regulation and socio-emotional processing. Further studies are needed to clarify the precise mechanisms and clinical implications of these associations.

Supplementary References

1. Price EM, Robinson WP. Adjusting for Batch Effects in DNA Methylation Microarray Data, a Lesson Learned. *Front Genet* 2018; **9:** 83.

2. Fortin JP, Triche TJ, Hansen KD. Preprocessing, normalization and integration of the Illumina HumanMethylationEPIC array with minfi. *Bioinformatics* 2017; **33**(4)**:** 558-560.

3. Smith AK, Ratanatharathorn A, Maihofer AX, Naviaux RK, Aiello AE, Amstadter AB *et al.* Epigenome-wide meta-analysis of PTSD across 10 military and civilian cohorts identifies methylation changes in AHRR. *Nat Commun* 2020; **11**(1)**:** 5965.

4. Katrinli S, Maihofer AX, Wani AH, Pfeiffer JR, Ketema E, Ratanatharathorn A *et al.* Epigenome-wide meta-analysis of PTSD symptom severity in three military cohorts implicates DNA methylation changes in genes involved in immune system and oxidative stress. *Mol Psychiatry* 2022; **27**(3)**:** 1720-1728.

5. Barfield RT, Kilaru V, Smith AK, Conneely KN. CpGassoc: an R function for analysis of DNA methylation microarray data. *Bioinformatics* 2012; **28**(9)**:** 1280-1281.

6. Leek JT, Johnson WE, Parker HS, Jaffe AE, Storey JD. The sva package for removing batch effects and other unwanted variation in high-throughput experiments. *Bioinformatics* 2012; **28**(6)**:** 882-883.

7. Tian Y, Morris TJ, Webster AP, Yang Z, Beck S, Feber A *et al.* ChAMP: updated methylation analysis pipeline for Illumina BeadChips. *Bioinformatics* 2017; **33**(24)**:** 3982-3984.

8. Teschendorff AE, Breeze CE, Zheng SC, Beck S. A comparison of reference-based algorithms for correcting cell-type heterogeneity in Epigenome-Wide Association Studies. *BMC Bioinformatics* 2017; **18**(1)**:** 105.

9. Fukunaga T, Mizoi Y, Yamashita A, Yamada M, Yamamoto Y, Tatsuno Y *et al.* Thymus of abused/neglected children. *Forensic Sci Int* 1992; **53**(1)**:** 69-79.

10. [Reports on medico-legal data from massive investigation performed by the Medico-Legal Society of Japan--weight and size of internal organs of normal Japanese today]. *Nihon Hoigaku Zasshi* 1992; **46**(3)**:** 225-235.

11. Suzuki S, Fujisawa TX, Sakakibara N, Fujioka T, Takiguchi S, Tomoda A. Development of Social Attention and Oxytocin Levels in Maltreated Children. *Sci Rep* 2020; **10**(1)**:** 7407.

12. Ochiai K, Nishitani S, Yao A, Hiraoka D, Kawata NYS, Suzuki S *et al.* Behavioral and emotional difficulties in maltreated children: Associations with epigenetic clock changes and visual attention to social cues. *PLoS One* In press.

13. Fujioka T, Fujisawa TX, Inohara K, Okamoto Y, Matsumura Y, Tsuchiya KJ *et al.* Attenuated relationship between salivary oxytocin levels and attention to social information in adolescents and adults with autism spectrum disorder: a comparative study. *Ann Gen Psychiatry* 2020; **19:** 38.

14. Ashburner J. A fast diffeomorphic image registration algorithm. *Neuroimage* 2007; **38**(1)**:** 95-113.

15. Ridgway GR, Omar R, Ourselin S, Hill DL, Warren JD, Fox NC. Issues with threshold masking in voxel-based morphometry of atrophied brains. *Neuroimage* 2009; **44**(1)**:** 99-111.

16. Nishitani S, Fujisawa TX, Hiraoka D, Makita K, Takiguchi S, Hamamura S *et al.* A multi-modal MRI analysis of brain structure and function in relation to OXT methylation in maltreated children and adolescents. *Transl Psychiatry* 2021; **11**(1)**:** 589.

17. Hüls A, Czamara D. Methodological challenges in constructing DNA methylation risk scores. *Epigenetics* 2020; **15**(1-2)**:** 1-11.

18. Naumova OY, Rychkov SY, Kornilov SA, Odintsova VV, Anikina V, Solodunova MY *et al.* Effects of early social deprivation on epigenetic statuses and adaptive behavior of young children: A study based on a cohort of institutionalized infants and toddlers. *PLoS One* 2019; **14**(3)**:** e0214285.

19. Teschendorff AE, Marabita F, Lechner M, Bartlett T, Tegner J, Gomez-Cabrero D *et al.* A beta-mixture quantile normalization method for correcting probe design bias in Illumina Infinium 450 k DNA methylation data. *Bioinformatics* 2013; **29**(2)**:** 189-196.

20. Horvath S. DNA methylation age of human tissues and cell types. *Genome Biol* 2013; **14**(10)**:** R115.

21. Cicchetti D, Hetzel S, Rogosch FA, Handley ED, Toth SL. An investigation of child maltreatment and epigenetic mechanisms of mental and physical health risk. *Dev Psychopathol* 2016; **28**(4pt2)**:** 1305-1317.

22. Papale LA, Seltzer LJ, Madrid A, Pollak SD, Alisch RS. Differentially Methylated Genes in Saliva are linked to Childhood Stress. *Sci Rep* 2018; **8**(1)**:** 10785.

23. Sumner JA, Gambazza S, Gao X, Baccarelli AA, Uddin M, McLaughlin KA. Epigenetics of early-life adversity in youth: cross-sectional and longitudinal associations. *Clin Epigenetics* 2022; **14**(1)**:** 48.

24. Yang BZ, Zhang H, Ge W, Weder N, Douglas-Palumberi H, Perepletchikova F *et al.* Child abuse and epigenetic mechanisms of disease risk. *Am J Prev Med* 2013; **44**(2)**:** 101-107.

25. Kumsta R, Marzi SJ, Viana J, Dempster EL, Crawford B, Rutter M *et al.* Severe psychosocial deprivation in early childhood is associated with increased DNA methylation across a region spanning the transcription start site of CYP2E1. *Transl Psychiatry* 2016; **6**(6)**:** e830.

26. Katrinli S, Oliveira NCS, Felger JC, Michopoulos V, Smith AK. The role of the immune system in posttraumatic stress disorder. *Transl Psychiatry* 2022; **12**(1)**:** 313.

27. Ohshima T, Nakaya T, Saito K, Maeda H, Nagano T. Child neglect followed by marked thymic involution and fatal systemic pseudomonas infection. *Int J Legal Med* 1991; **104**(3)**:** 167-171.

28. Sabatini DM. Twenty-five years of mTOR: Uncovering the link from nutrients to growth. *Proc Natl Acad Sci U S A* 2017; **114**(45)**:** 11818-11825.

29. Brauer AU, Savaskan NE, Kuhn H, Prehn S, Ninnemann O, Nitsch R. A new phospholipid phosphatase, PRG-1, is involved in axon growth and regenerative sprouting. *Nat Neurosci* 2003; **6**(6)**:** 572-578.

30. Bremner JD, Staib LH, Kaloupek D, Southwick SM, Soufer R, Charney DS. Neural correlates of exposure to traumatic pictures and sound in Vietnam combat veterans with and without posttraumatic stress disorder: a positron emission tomography study. *Biol Psychiatry* 1999; **45**(7)**:** 806-816.

31. Rolls ET. The cingulate cortex and limbic systems for emotion, action, and memory. *Brain Struct Funct* 2019; **224**(9)**:** 3001-3018.

32. Kanwisher N, Yovel G. The fusiform face area: a cortical region specialized for the perception of faces. *Philos Trans R Soc Lond B Biol Sci* 2006; **361**(1476)**:** 2109-2128.

33. Gobbini MI, Leibenluft E, Santiago N, Haxby JV. Social and emotional attachment in the neural representation of faces. *Neuroimage* 2004; **22**(4)**:** 1628-1635.

34. Ramon M, Dricot L, Rossion B. Personally familiar faces are perceived categorically in face-selective regions other than the fusiform face area. *Eur J Neurosci* 2010; **32**(9)**:** 1587-1598.

35. Takashima A, van der Ven F, Kroes MC, Fernández G. Retrieved emotional context influences hippocampal involvement during recognition of neutral memories. *Neuroimage* 2016; **143:** 280-292.

36. Vogt BA, Laureys S. *The primate posterior cingulate gyrus: Connections, sensorimotor orientation, gateway to limbic processing*. Oxford University Press2009.

37. Cheng W, Rolls ET, Qiu J, Xie X, Wei D, Huang CC *et al.* Increased functional connectivity of the posterior cingulate cortex with the lateral orbitofrontal cortex in depression. *Transl Psychiatry* 2018; **8**(1)**:** 90.

38. Phillips ML, Ladouceur CD, Drevets WC. A neural model of voluntary and automatic emotion regulation: implications for understanding the pathophysiology and neurodevelopment of bipolar disorder. *Mol Psychiatry* 2008; **13**(9)**:** 829, 833-857.

39. Cavalcante IP, Berthon A, Fragoso MC, Reincke M, Stratakis CA, Ragazzon B *et al.* Primary bilateral macronodular adrenal hyperplasia: definitely a genetic disease. *Nat Rev Endocrinol* 2022; **18**(11)**:** 699-711.

40. Alink LR, Cicchetti D, Kim J, Rogosch FA. Longitudinal associations among child maltreatment, social functioning, and cortisol regulation. *Dev Psychol* 2012; **48**(1)**:** 224-236.

41. Mizushima SG, Fujisawa TX, Takiguchi S, Kumazaki H, Tanaka S, Tomoda A. Effect of the Nature of Subsequent Environment on Oxytocin and Cortisol Secretion in Maltreated Children. *Front Psychiatry* 2015; **6:** 173.

42. Tomoda A, Navalta CP, Polcari A, Sadato N, Teicher MH. Childhood sexual abuse is associated with reduced gray matter volume in visual cortex of young women. *Biol Psychiatry* 2009; **66**(7)**:** 642-648.

43. Fujisawa TX, Shimada K, Takiguchi S, Mizushima S, Kosaka H, Teicher MH *et al.* Type and timing of childhood maltreatment and reduced visual cortex volume in children and adolescents with reactive attachment disorder. *Neuroimage Clin* 2018; **20:** 216-221.
