# Supplementary material for: Multi-epigenome-wide analyses and meta-analysis of child maltreatment in judicial autopsies and intervened children and adolescents
Source: Mol Psychiatry. 2025 Sep 16;31(3):1253–64. doi: 10.1038/s41380-025-03236-1 (PMC12916479; doi:10.1038/s41380-025-03236-1)
Supplement: Supplementary file 4 — Supplementary Figures [file 41380_2025_3236_MOESM4_ESM.pdf]

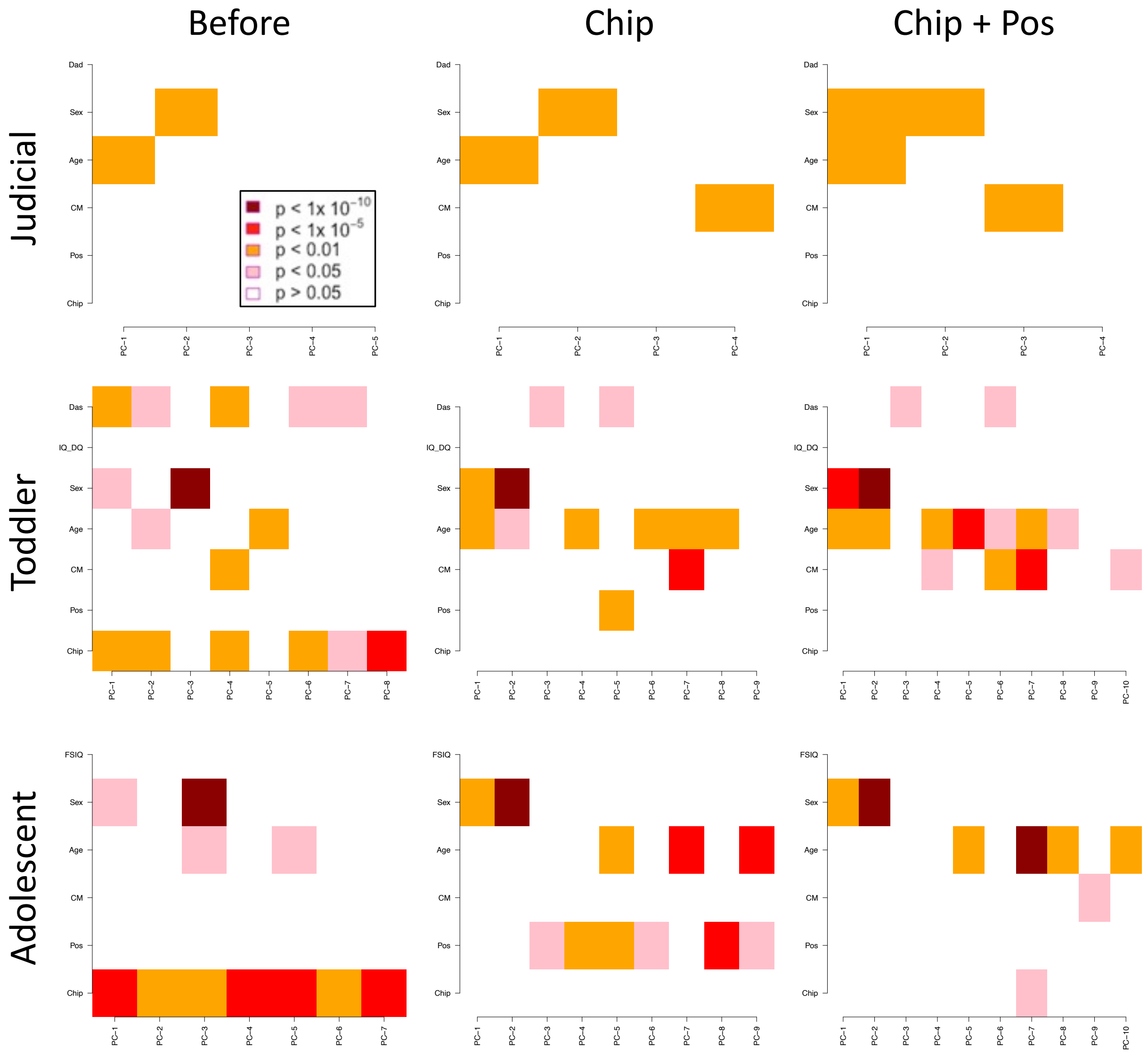

Supplementary Figure S1. Singular Value Decomposition (SVD) analysis before and after ComBat adjustments to remove Chip and positional (Pos) batch effects. Group (CM and TD), age, and sex were retained during ComBat adjustments.

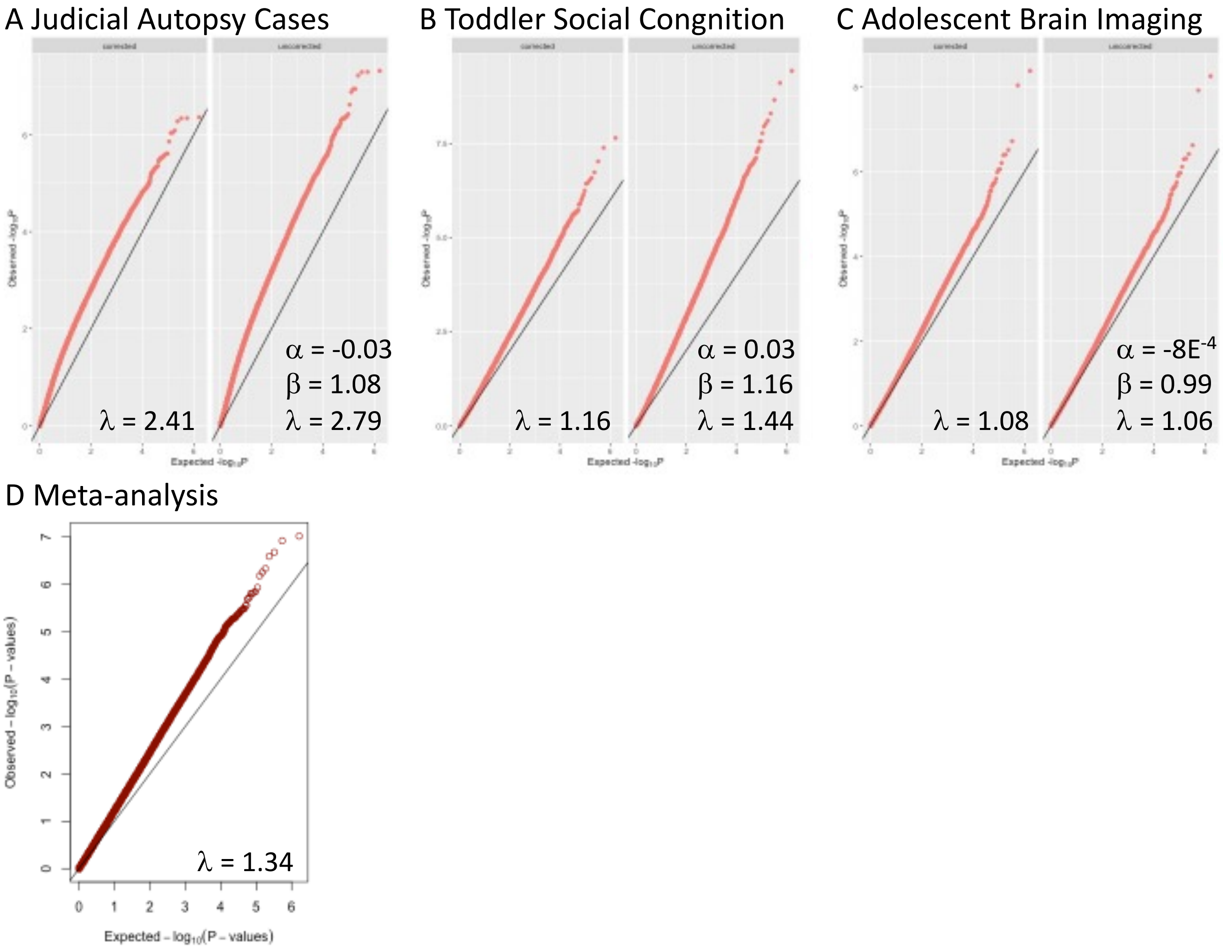

Supplementary Figure S2. Q-Q plots for the EWAS of (A) Judicial Autopsy Cases, (B) Toddler Social Cognition, and (C) Adolescent Brain Imaging cohorts, as well as for the corresponding (D) meta-analysis. For each of these three datasets, the left panel shows the distribution after bacon correction, and the right panel shows the distribution before bacon correction. α indicates the average shift estimated by the bacon package, β indicates the inflation factor estimated by the bacon package, and λ indicates the genomic inflation factor.

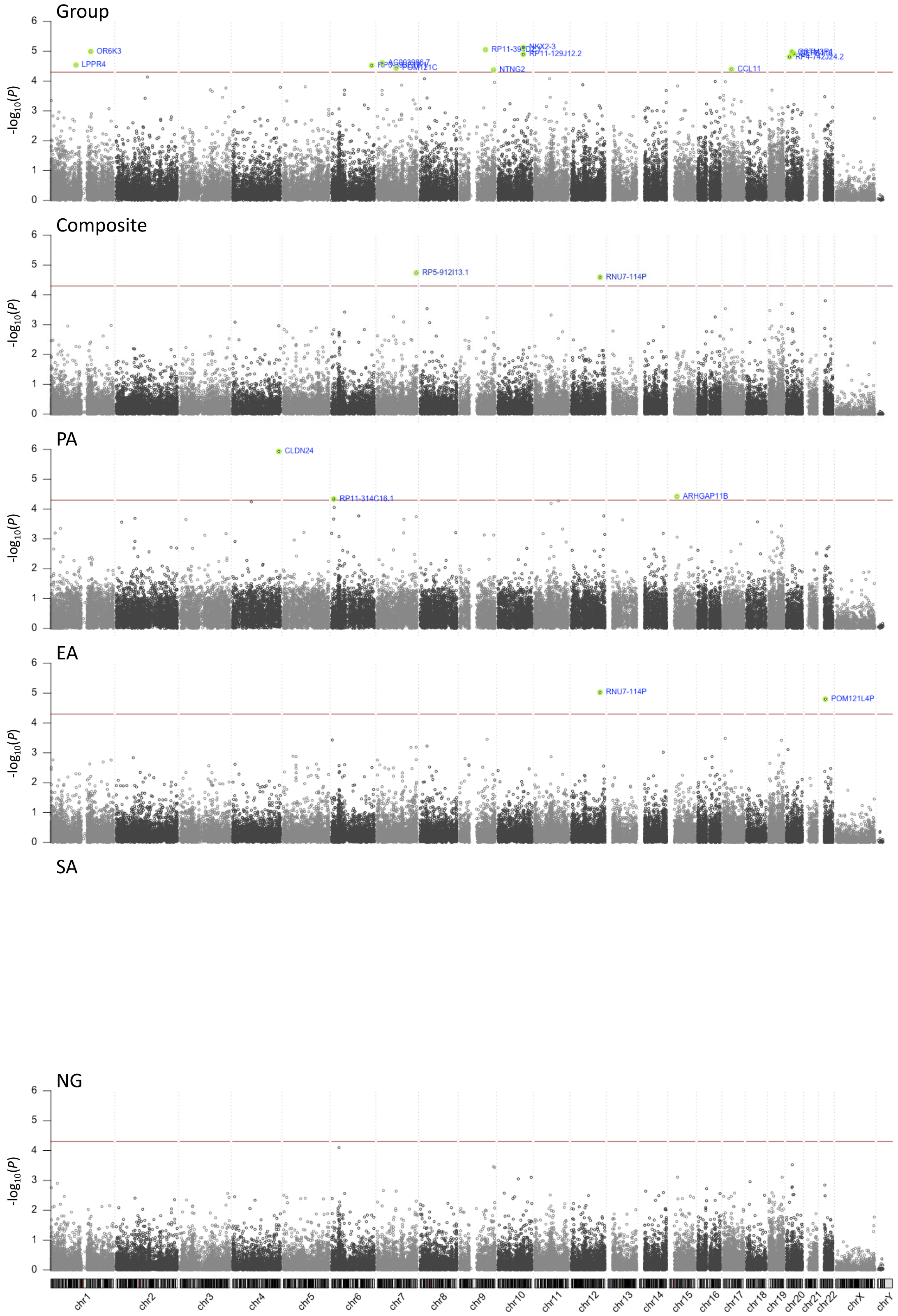

Supplementary Figure S3. GAMuT Manhattan plot for the association between DNA methylation and maltreatment type across all and individual domains in the Toddler Social Cognition cohort. Red line: *P*-value < 5.0E-05

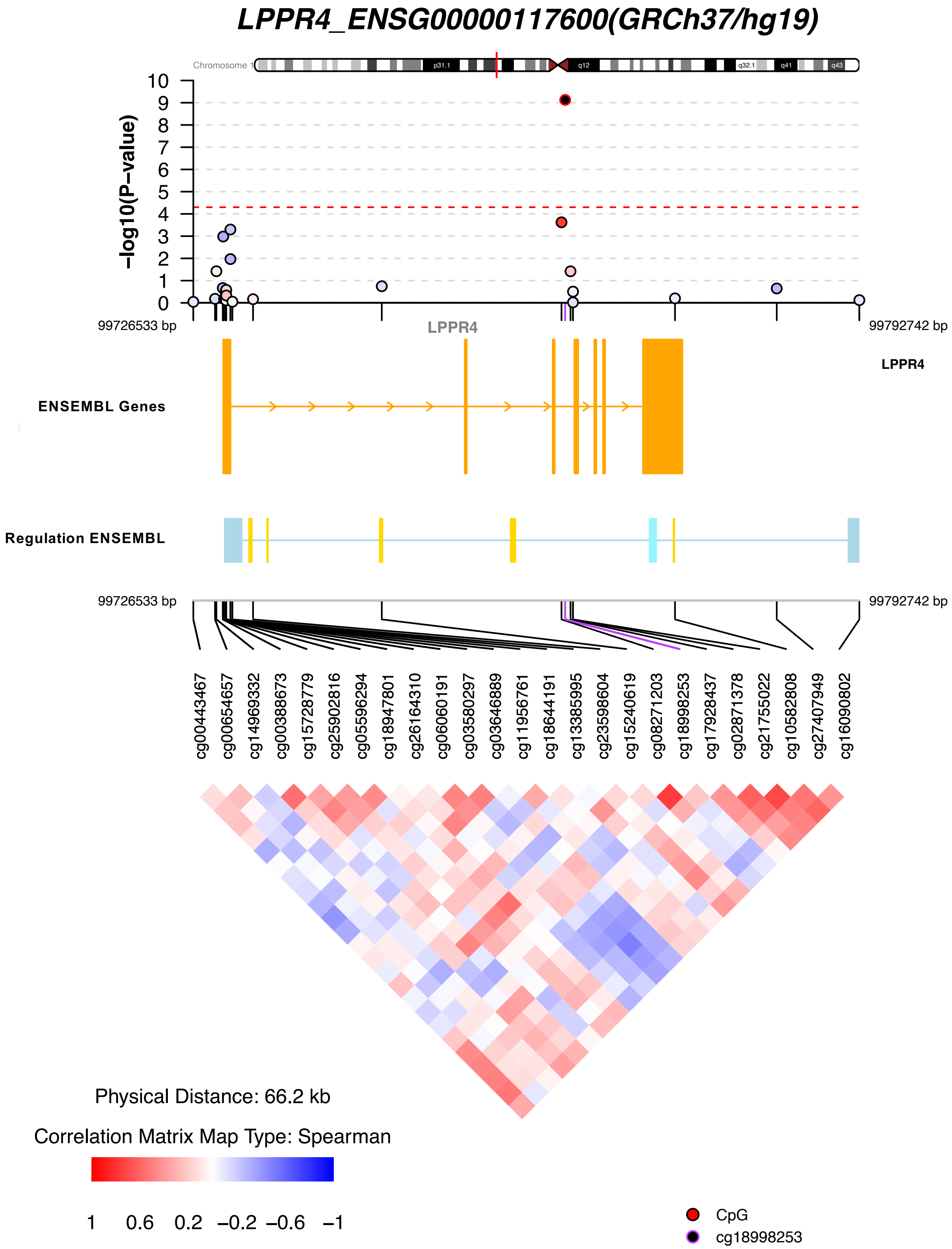

Supplementary Figure S4. Association of 25 CpG sites across the *LPPR4* gene with child maltreatment in the Toddler Social Cognition cohort. The top probe: cg18998253.

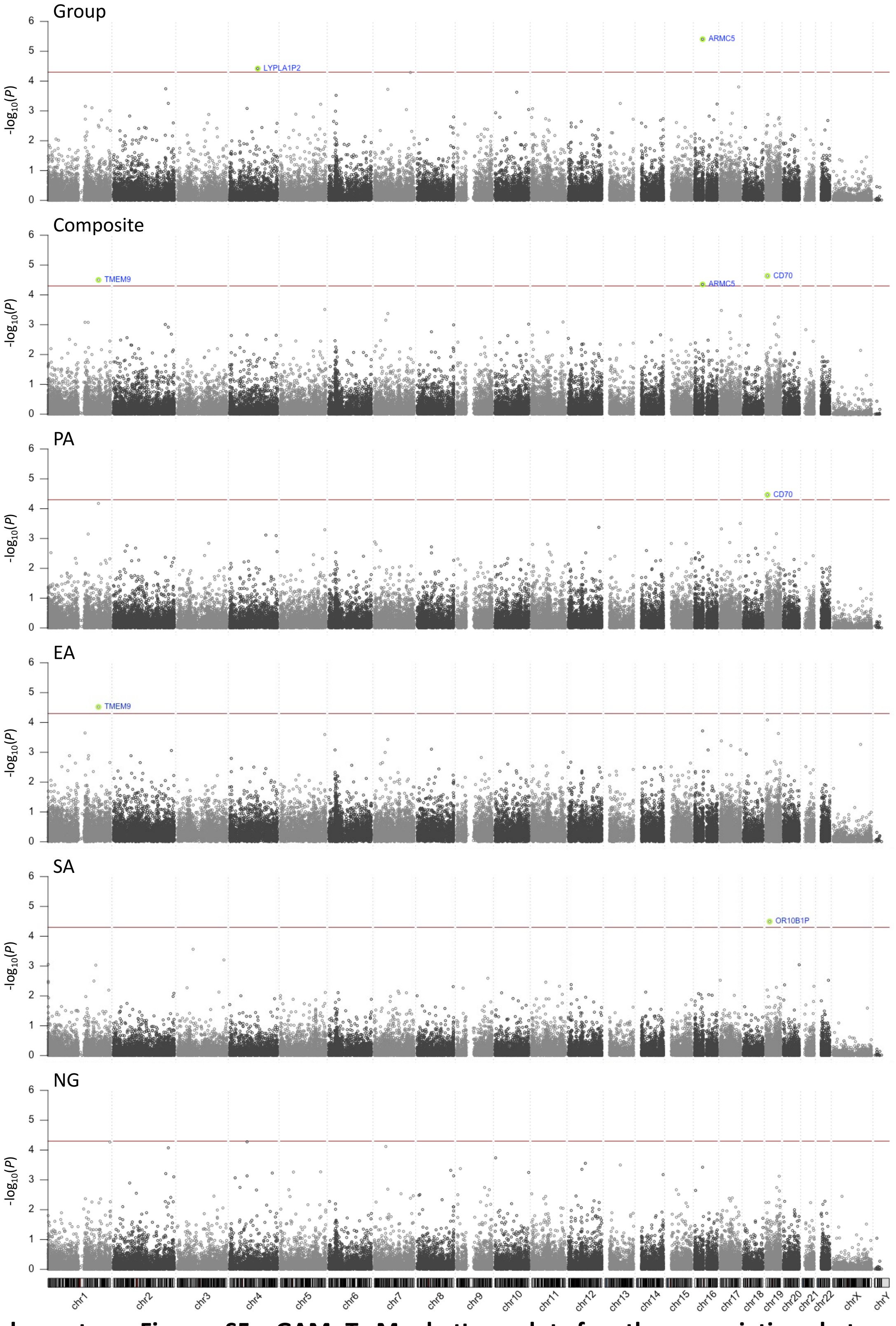

Supplementary Figure S5. GAMuT Manhattan plot for the association between DNA methylation and maltreatment type across all and individual domains in the Adolescent Brain Imaging cohort. Red line: *P*-value < 5.0E-05

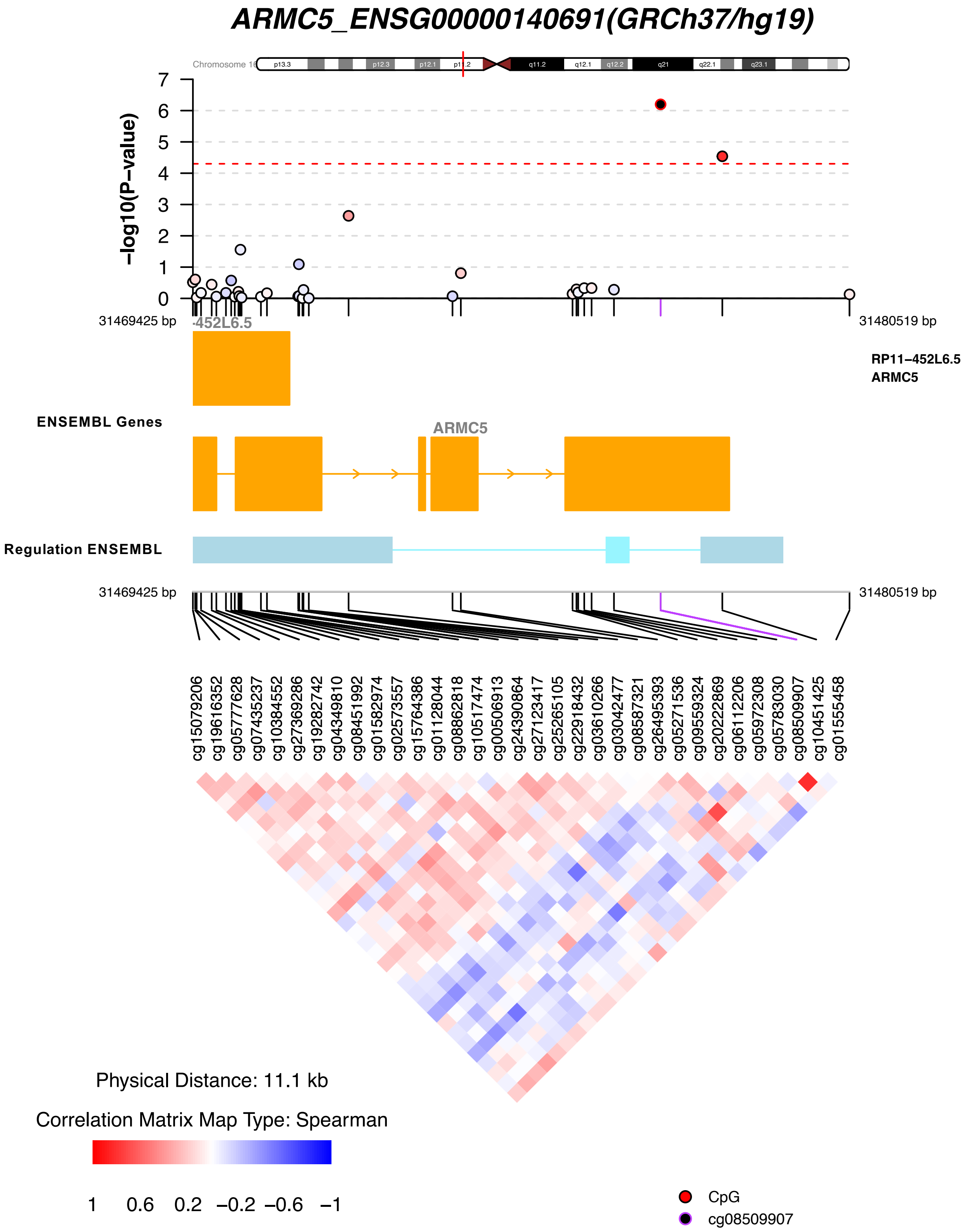

Supplementary Figure S6. Association of 33 CpG sites across the *ARMC5* gene with child maltreatment in the Adolescent Brain Imaging cohort. cg08587321: the top probe.

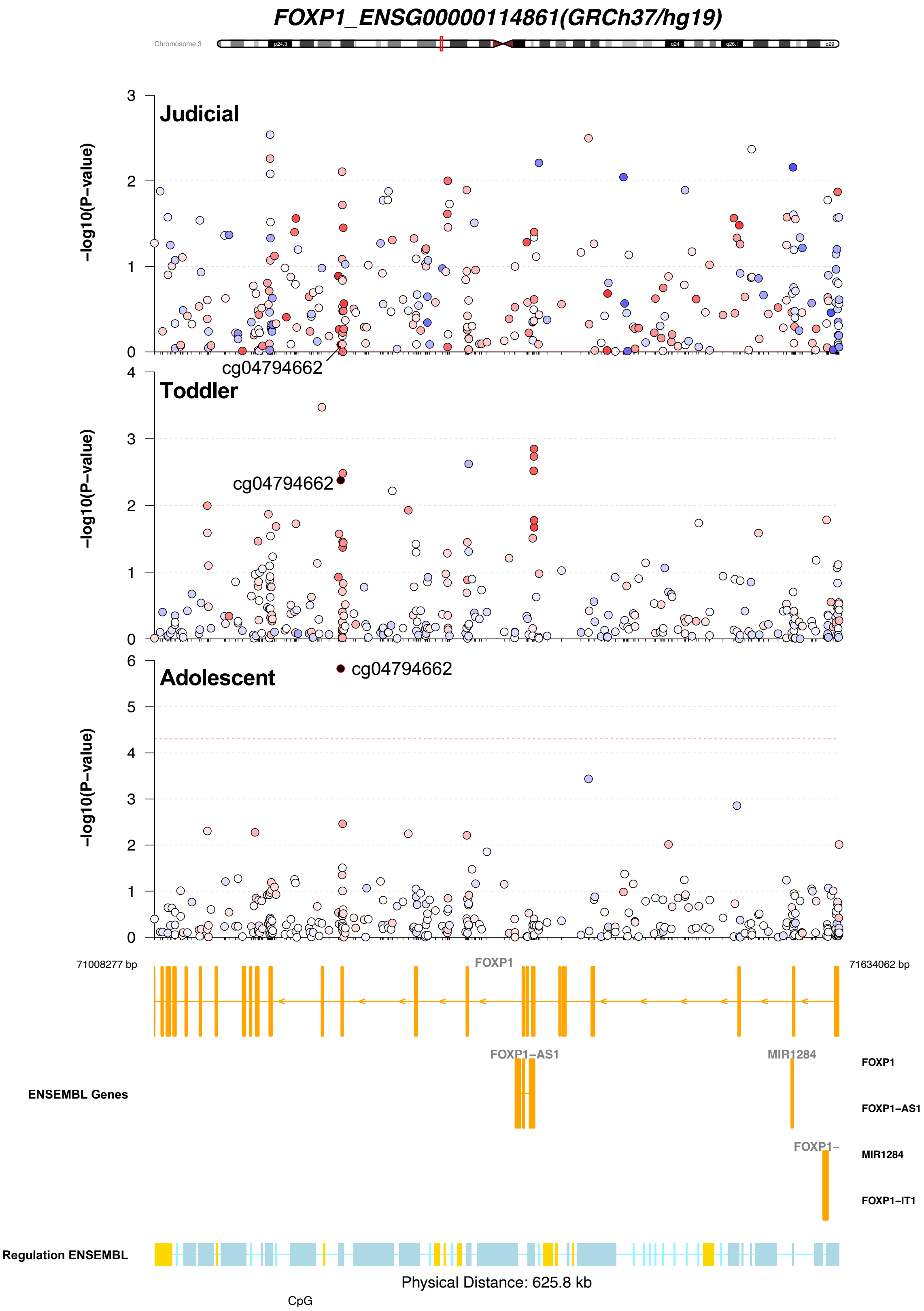

Supplementary Figure S7. Association of 297 CpG sites across the *FOXP1* gene with child maltreatment in the three cohorts. cg04794662: the top probe.

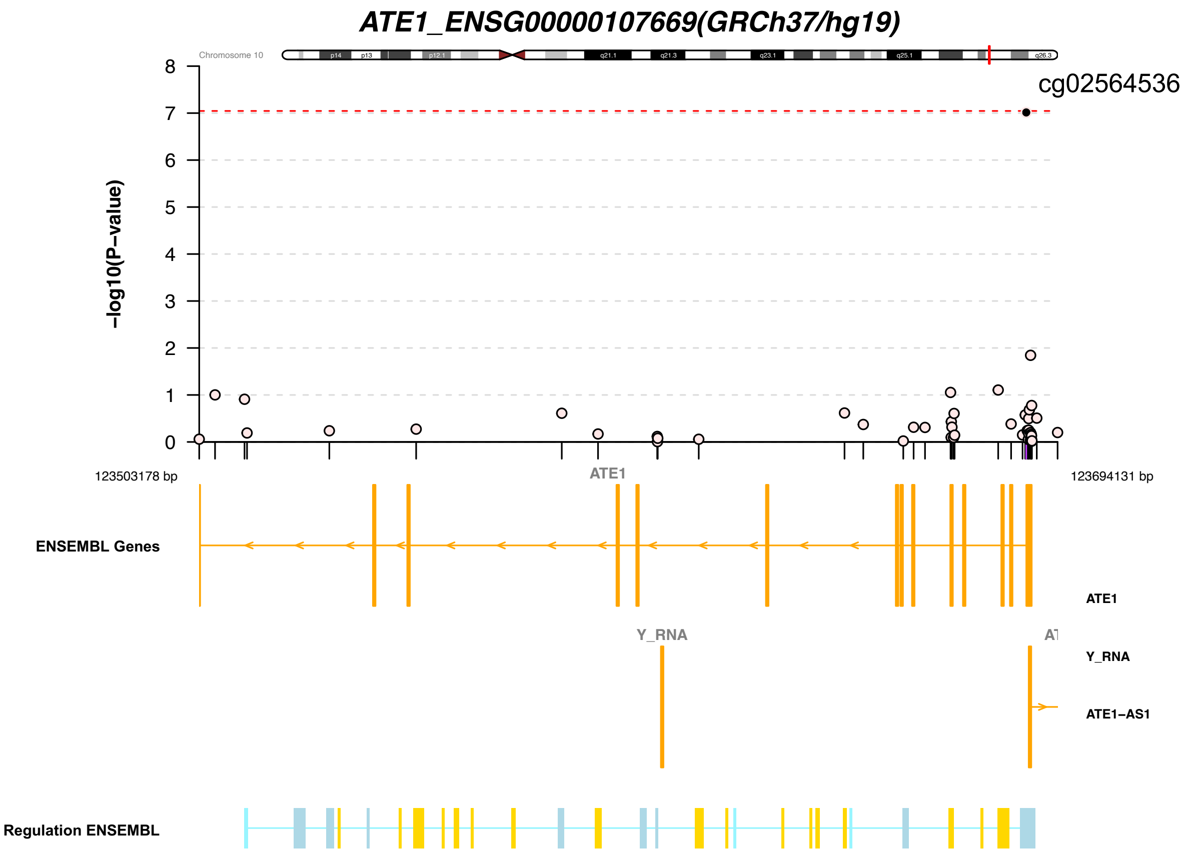

Supplementary Figure S8. Association of 46 CpG sites across the *ATE1* gene with child maltreatment in the meta-analysis. cg02564536: *q*-value significant CpG.

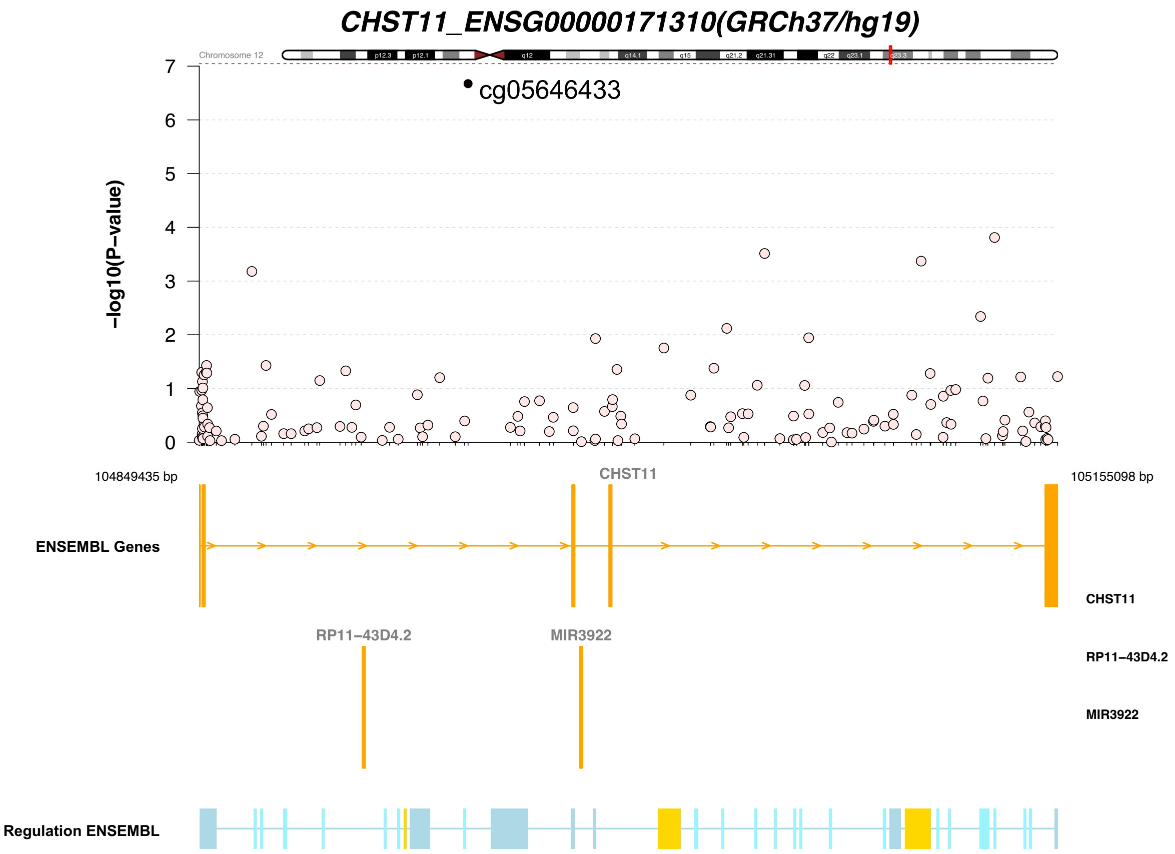

Supplementary Figure S9. Association of 142 CpG sites across the *CHST11* gene with child maltreatment in the meta-analysis. cg05646433: *q*-value significant CpG.

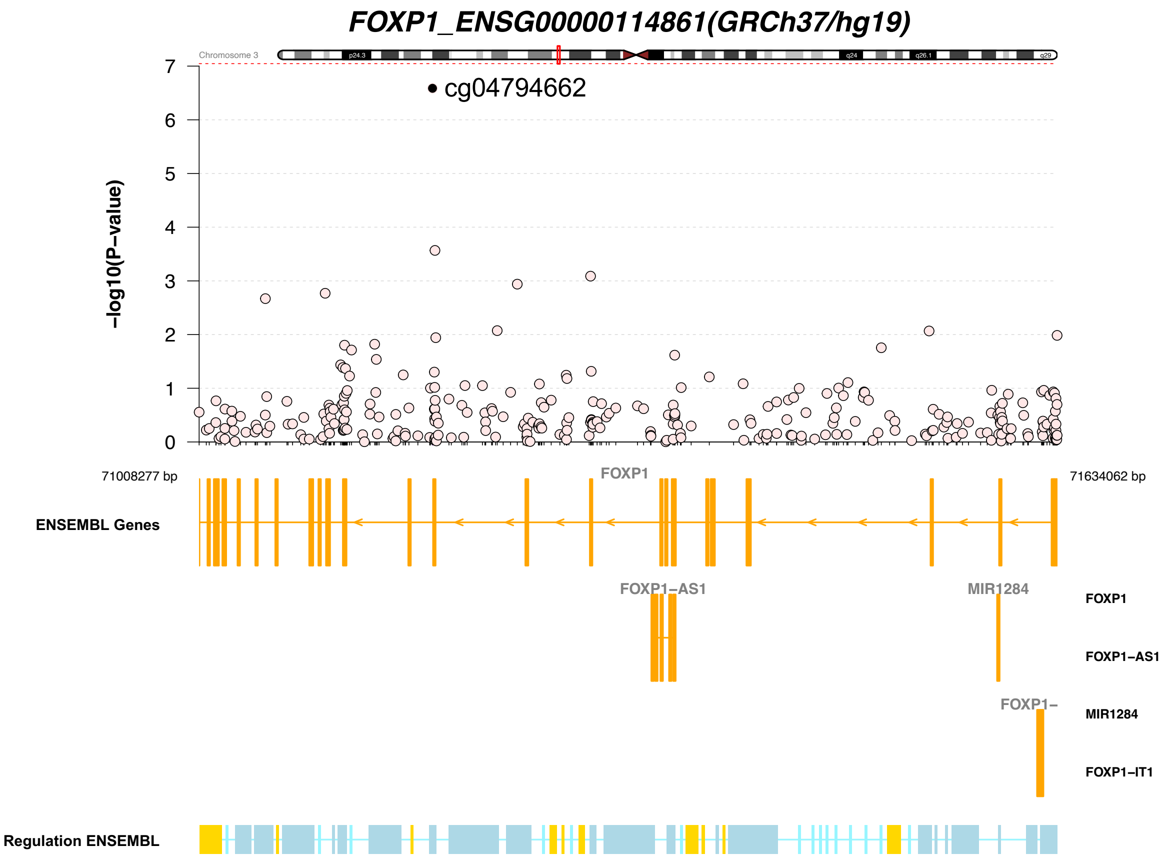

Supplementary Figure S10. Association of 297 CpG sites across the *FOXP1* gene with child maltreatment in the meta-analysis. cg04794662: *q*-value significant CpG.

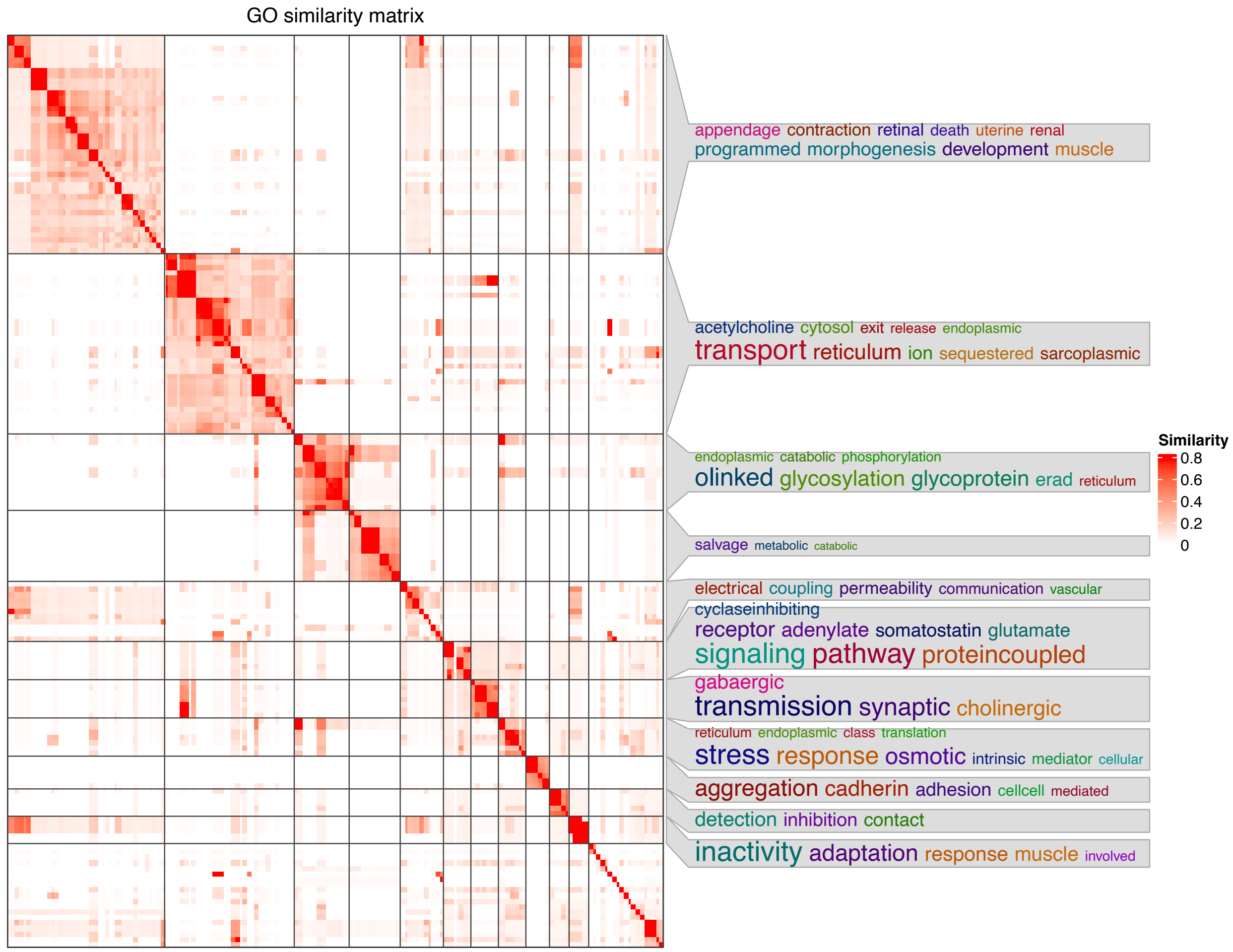

Supplementary Figure S11. Gene Ontology (GO) semantic similarity matrix for significant 167 GO enrichment BP results. Left: the semantic similarity matrix heatmap, Right: the word cloud annotations which summarize the functions with keywords in each GO cluster.

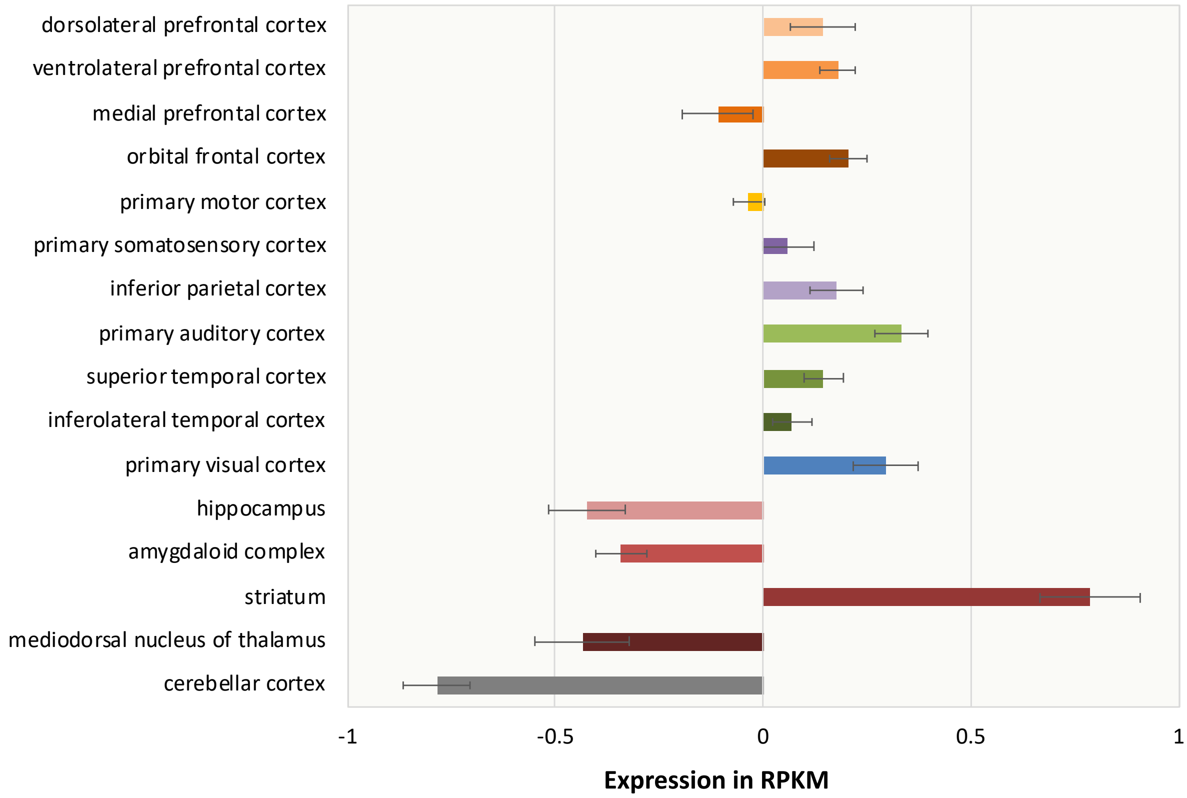

Supplementary Figure S12. *FOXP1* gene expression in the developing human brain. The figure shows the *FOXP1* gene expression measured by RNA-seq in the BrainSpan (http://www.brainspan.org) datasets (n = 15; mean age: 11.9 ± 8.9; age range: 1-30 years). Error bars indicate the standard error of the mean.
